# Ploidy-Seq: inferring mutational chronology by sequencing polyploid tumor subpopulations

**DOI:** 10.1186/s13073-015-0127-5

**Published:** 2015-01-28

**Authors:** Ankit Malhotra, Yong Wang, Jill Waters, Ken Chen, Funda Meric-Bernstam, Ira M Hall, Nicholas E Navin

**Affiliations:** The Jackson Laboratory for Genomic Medicine, Farmington, CT USA; Department of Genetics, MD Anderson Cancer Center, Houston, TX USA; Department of Bioinformatics, MD Anderson Cancer Center, Houston, TX USA; Department of Surgical Oncology, MD Anderson Cancer Center, Houston, TX USA; Department of Investigational Cancer Therapeutics, MD Anderson Cancer Center, Houston, TX USA; The Genome Institute, Washington University School of Medicine, St. Louis, MO USA; Department of Medicine, Washington University School of Medicine, St. Louis, MO USA; Graduate Program in Genes and Development, Graduate School of Biomedical Sciences, University of Texas Health Science Center, Houston, TX USA

## Abstract

Human cancers are frequently polyploid, containing multiple aneuploid subpopulations that differ in total DNA content. In this study we exploit this property to reconstruct evolutionary histories, by assuming that mutational complexity increases with time. We developed an experimental method called Ploidy-Seq that uses flow-sorting to isolate and enrich subpopulations with different ploidy prior to next-generation genome sequencing. We applied Ploidy-Seq to a patient with a triple-negative (ER-/PR-/HER2-) ductal carcinoma and performed whole-genome sequencing to trace the evolution of point mutations, indels, copy number aberrations, and structural variants in three clonal subpopulations during tumor growth. Our data show that few mutations (8% to 22%) were shared between all three subpopulations, and that the most aggressive clones comprised a minority of the tumor mass. We expect that Ploidy-Seq will have broad applications for delineating clonal diversity and investigating genome evolution in many human cancers.

**Electronic supplementary material:**

The online version of this article (doi:10.1186/s13073-015-0127-5) contains supplementary material, which is available to authorized users.

## Background

Genome evolution is challenging to study in human patients, because we cannot ethically sample the patient at multiple time points during the progression of the disease. Even when samples can be obtained at different time points [1-3], the tumor genome has usually been remodeled by chemotherapy, making it difficult to observe its natural course of evolution. An alternative approach is to reconstruct genome evolution *ex post facto* from a single tumor sample. As tumor cells evolve, they form distinct lineages and subpopulations as they mutate and encounter selective pressures, resulting in intratumor heterogeneity. While clonal diversity is considered ‘bad news’ from a clinical standpoint, it provides a permanent record of the mutations that occur during tumor evolution. By assuming that mutational complexity increases over time, we can use mutations as stable markers of evolution to reconstruct the relative chronology of the mutations during the natural history of the disease.

In our previous work, we reconstructed the evolution of breast cancer genomes using copy number alterations (CNAs) as stable markers of evolution [4,5]. Several other approaches have also been developed to resolve intratumor genomic heterogeneity. In several studies, next-generation sequencing was performed on bulk tumors to measure mutation frequencies, which were normalized by copy number to estimate tumor subpopulation fractions [3,6,7]. While this approach can resolve some population substructure, it is inherently unable to distinguish which combinations of mutations co-exist in any particular subpopulation. Another approach involves spatially sampling distinct regions within a tumor for exome sequencing to reconstruct the evolutionary lineage of the tumor cells [8-10]. While this approach can resolve spatially segregated subpopulations, it cannot resolve multiple subpopulations that are intermixed in single regions within the tumor. An alternative approach is to use single cell sequencing methods [11-14], which have the capability of fully resolving admixtures of genetically distinct clones. While powerful, these methods are currently limited by the number of cells that can be analyzed due to considerable associated costs. Due to these technical and economical limitations, the clonal diversity and patterns of genome evolution still remain poorly understood in most human cancers.

Triple-negative (ER-/PR-/Her2-) breast cancers (TNBCs) are particularly amenable for lineage tracing studies, since they display extensive intratumor genomic heterogeneity [7] and show very high numbers of genomic mutations [7,11,15]. These mutations can serve as stable markers of evolution for reconstructing the natural history of the tumor. TNBCs are also of considerable interest to the clinical community, because they have the poorest 5-year survival rates and cannot be treated with many targeted or hormonal therapies [16,17]. Thus, there is a strong interest in improving our fundamental understanding of how these tumors evolve genomic mutations and generate intratumor heterogeneity that confounds clinical diagnostics and treatment of patients.

## Methods

### Tumor sample

The de-identified frozen breast tumor sample was obtained from the Cooperative Human Tissue Network (CHTN). Genomic data from this tumor sample were reported in two previous studies [4,11]. Histopathological analysis classified the tumor as an invasive ductal carcinoma, Richardson-bloom grade (III) with significant immune cell infiltration and poor differentiation. The tumor sample stained negative for estrogen receptor, progesterone receptor and Her2 receptor by immunohistochemistry, and was therefore classified as triple-negative (ER-/PR-/Her2-).

### Human subjects

No human subjects participated in this study. The ductal carcinoma sample was obtained as a de-identified frozen tumor specimen from the Cooperative Human Tissue Network (CHTN). This study was approved by the Cold Spring Harbor IRB regulatory committee on 15 June 2010 under exemption 45 CFR.46.101(b)(4).

### Data access

The data from this study have been uploaded to the Sequence Read Archive (NCBI) and are available for download under accession SRP013572.

### Ploidy-Seq

Nuclei were isolated from cell lines and from the frozen tumor using an NST-DAPI buffer and 0.1% DNase-free RNase A. The frozen tumor sample was macrodissected and nuclei were isolated from two regions (R1 and R2) for FACS by finely mincing a tissue in a Petri dish in 1.0 to 2.0 mL of NST-DAPI buffer. The nuclear suspensions were filtered through 37-μm plastic mesh prior to flow-sorting. Prior to FACS we performed cytometric analysis of ploidy distributions using the LSRII system (BD Biosciences). A small amount of prepared nuclei from each tumor sample was mixed with a diploid control sample (derived from a lymphoblastoid cell line of an apparently normal person) to accurately determine the diploid peak position within the tumor DNA content distribution. Nuclei were subsequently flow-sorted on the AriaII (BD Biosciences) by gating cellular distributions with differences in their total genomic DNA content according to DAPI intensity. Nuclei from each ploidy distribution (1.7 N, 2 N, 3.1 N, and 3.3 N) were collected in separate 1.5 mL tubes and DNA was isolated and purified using the Qiagen QiaAMP mini kit (cat #51304).

### Next-generation sequencing

We acoustically sonicated 1ug of purified DNA to 200 to 300 bp using the Covaris Sonicator S220. Libraries were constructed using NEBNext DNA library Prep Master Mix Set for Illumina (New England Laboratory, #F6040L) for end-repair, 3′adenylation and ligation according to manufacturer’s instructions. The MinElute PCR Purification Kit (Qiagen, #28006) was used for the purification steps during library prep. After agarose electrophoresis the 250 to 350 bp fraction was excised for purification. We then performed eight cycles of PCR following manufacturer’s direction, using PE5/7 primers (Illumina Inc). Agencourt AMPure XP (Beckman Coulter, #A63881) was used for final purification. Final concentration was measured by quantitative PCR using the KAPA Library Quantification Kit (KAPA Biosystems, KK4835) and ABI PRISM real-time machine (Applied Biosystem 7900HT) and the size distribution was determined using the 2100 Bioanalyzer (Agilent). Each library was run at 100 single-end cycles on the Illumina HiSeq2000 or GA2 systems using four to eight flow cell lanes. Data was processed using the CASAVA 1.8.1 pipeline (Illumina Inc.) and sequence reads were converted to FASTQ files.

### Data processing and alignment

Sequence reads in FASTQ format were mapped to the human assembly US National Center for Biotechnology Information (NCBI) build 36 (hg18) using the Burrows-Wheeler alignment tool (BWA version 0.6.0) [18] with default parameters and sample option to create SAM files with correct mate pair information, with a read group tag that includes the sample name. We then used Samtools (0.1.16) [19] to convert SAM files to compressed BAM files and sort the BAM files by chromosome coordinate. The Genome Analysis Toolkit (GATK v1.4-37) was used to locally realign the BAM files at intervals that may have insertion/deletion (indel) alignment errors before PCR duplicate marking with Picard (version 1.56) [20].

### Single nucleotide and indel variant detection

We used GATK UnifiedGenotyper to detect single nucleotide variants (SNVs) and small indels [21]. We then used the GATK variant recalibrator to filter the output at default sensitivity level. Recalibration training sets included hapmap 3.3, dbSNP build 132, Omni 2.5 M chip, and Mills. Annotations used for training included variant quality score by depth (QD), mapping quality rank sum score, read position rank sum score, mapping quality (MQ), coverage depth (DP), and strand bias (FS). We required a minimum base quality (mbq) of 20 for the base to be considered during variant detection. Coverage depth at a given locus of greater than 2,500 reads was down sampled. All tumor subpopulation samples (H, AA, and AB) were processed together with the Diploid (D) sample to generate a single VCF4 file. Somatic variants were distinguished from germline variants by excluding mutations sites present in the Diploid sample. We then used GATK SelectVariants to separate SNVs and indels into two VCF4 files for downstream annotation. During variant calling we required a minimum coverage depth of 20 and minimum number of variants reads of 5 to call SNVs or indels.

### Copy number detection

Copy number alterations were detected from read depth data using the variable binning method [12]. Briefly, copy number is calculated from read density, by dividing the genome into ‘bins’ and counting the number of unique reads in each bin. To determine interval sizes we simulated sequence reads by sampling 200 million sequences of length 48 from the human reference genome (HG18/NCBI36) and introduced single nucleotide errors with a frequency typically encountered during Illumina sequencing. These sequences were mapped back to the human reference genome using BWA and filtered for unique mappings. We assigned a number of bins to each chromosome based on the proportion of simulated reads mapped. We then divided each chromosome into bins with an equal number of simulated reads. This resulted in 50,009 genomic bins with no bins crossing chromosome boundaries. The median genomic length spanned by each bin is 54 kb. This variable binning efficiently reduces false deletion events when compared to uniform length-fixed bins. We then applied Loess normalization to correct for GC bias. The copy number profiles were then segmented using the Kolmogorov-Smirnov (KS) statistical test [22].

### Detection of structural variants by split read mapping (SRM)

Paired-end reads (readpairs) were aligned to the reference genome (hg18) using *NOVOALIGN* with an index word size of 14 and step size of 1 (−k 14 -s 1) using ‘Random’ mode with empirically estimated insert size and standard deviation. We extracted all readpairs where one or more read did not align to the reference genome, or where one or more read had been soft-clipped by at least 25 bp. Since the reads could be unmapped or soft-clipped due to poor quality, we removed all readpairs that had more than 4% N bases in either of the reads. The remaining readpairs were written to fastq files and fed into the FLASH program [23] to generate larger contigs from overlapping reads (−f < mean insert size > −s < insert size standard deviation > −r 100). All merged and unmerged reads were aligned to the reference genome using BWA-SW [24] using sensitive settings (−z 10 -H). Split-read mappings with at least 25-bp of non-overlap with an adjacent mapping on the query sequence were extracted from the BAM file, converted to BEDPE format [25] and subjected to strict duplicate removal using *dedupDiscordantsMultiPass* (−s 3).

Split-read mappings were clustered into breakpoint calls using a custom algorithm (SRM). SRM first converts split-read mappings into predicted breakpoint intervals (+/− 1-bp) and clusters intervals where both ends overlap one another and predict the same variant type (for example, deletion). SRM then performs an ‘all by all’ comparison of all split-read mappings within each cluster to identify the breakpoint that has the most supporting split-reads. This breakpoint is reported as representative of the cluster. In order to maximize the sensitivity of presence/absence genotyping, reads from all samples (for example, AA, AB, H, and D) were combined before clustering, and after clustering the number of reads from each sample were counted to determine patterns of SV sharing among the samples.

To reduce false positives arising due to read mapping and library construction artifacts, breakpoint calls were filtered using the following criteria: (1) the breakpoint was defined by at least three reads; (2) the breakpoint call was at least 50-bp in size; (3) neither of the two breakpoints overlapped a simple sequence or satellite repeat by more than 50%, as determined by running *bedtools pairToBed* (−type either -f 0.5) against a union of the UCSC ‘simpleRepeat’ track and both simple and satellite repeat annotations from the ‘RepeatMasker’ track; (4) the mean mapping quality of the clustered mappings was greater than 30; and (5) the split-read mappings identifying a breakpoint were staggered by a total of at least 3-bp on the non-breakpoint end. This last step was done to remove false positives from multiple exact matching reads from different samples (which can occur due to alignment artifacts).

### Detection of structural variants with CREST

We ran the CREST pipeline [26] using default settings on each of the subpopulation (AA, AB, H, and D) bam files, which were generated by alignment with BWA. The pipeline involves four steps. First, putative breakpoints indicated by at least two soft-clipped reads were extracted from each chromosome. Second, putative somatic breakpoints in each of the three cancer genomes were generated by subtracting those that were also detected in the matched normal genome. Third, breakpoint sequences were assembled by CAP3 [27] using soft-clipped reads, and were aligned to the reference genome using BLAT to identify split-read mappings. A breakpoint was called if a pair of identical breakpoint positions were identified from the BLAT alignment of two distinct assembled breakpoint sequences. Calls obtained by CREST and SRM were combined by comparing their coordinates and variant types. Variants of the same type where both ends overlapped one another (+/− 10 bp) were considered to be due to the same underlying somatic mutation, and the call from SRM was retained.

### SV breakpoint assembly, validation, and genotyping

To validate breakpoints from SRM and CREST we performed *de novo* breakpoint assembly precisely as in our previous study [28,29] using a modified version of the SGA assembler. Briefly, we modified the *sga walk* function to report all walks from all connected components of the string graph. This modification allows for efficient assembly of low frequency somatic variants. For each breakpoint predicted by SRM or CREST, we extracted readpairs that mapped within 500-bp of the predicted breakpoints, including readpairs with one unmapped read. We then ran the following assembly pipeline: *sga preprocess* (default), *sga index* (−−no-reverse), *sga correct* (−k 13 -x 2 -d 128), *sga index* on the error corrected reads (default), *sga rmdup* (default), *sga overlap* (−m 15), *sga assemble* (−m 15 -d 0 -g 0 -b 0 -l 100), and our modified version of *sga walk* (−d 10000 --component-walks). Resulting contigs were aligned to the reference genome using BWA-SW (v.0.5.9) [24]. Split-mappings with at least 25 bp of non-overlap with an adjacent mapping on the query sequence were extracted from the BAM file and converted to BEDPE format, where each predicted breakpoint was represented by a 3-bp interval. We then used *pairToPair* (−type both) from the BEDTools software suite [25] to assess whether the original breakpoint calls were validated by split-mapping contigs. We considered a call to be validated if the breakpoints predicted by *de novo* assembly overlapped with the original breakpoints predicted by SRM/CREST and were of the same variant class (for example, deletion). We next performed a more sensitive breakpoint genotyping step in order to exclude germline SV breakpoints that may have been misclassified as somatic mutations due to inadequate coverage in the diploid (D) sample, and to obtain more accurate patterns of mutation sharing among the tumor subpopulations. We aligned all of the raw reads from each of the four samples to the library of breakpoint-containing contigs using BWA (default options). We considered a breakpoint to be present in the D sample (and thus to be a germline SV) if one or more reads from the D sample aligned to the breakpoint-containing contig with at least 20 aligned bases on either side of the novel junction. For the resulting somatic breakpoints that were not detected in D, we determined their presence/absence among the three tumor subpopulations using the same breakpoint contig alignment strategy as in the D line, except that we required at least two aligned reads to produce a positive genotype.

Finally, calls obtained by CREST and SRM were combined by comparing their coordinates and variant types. Variants of the same type where both ends overlapped one another (+/− 10 bp) were considered to be due to the same underlying somatic mutation, and the call from SRM was retained.

### Detection of loss of heterozygosity (LOH)

To map LOH events we first defined a set of 1,386,955 high confidence heterozygous germline SNPs from the GATK callset using the following criteria: (1) the SNP was determined by GATK to have a heterozygous genotype in the normal diploid (D) sample; and (2) the alternate SNP allele was present at a frequency of 0.25 to 0.75 in the D sample, as determined by counting the number of aligned reads that contained the alternate versus the reference SNP allele. At each of these SNPs and for each of the three tumor samples (AA, AB, and H) we then calculated the minor allele frequency (MAF), which is defined as the minimum of the alternate and reference allele frequencies. We measured the mean MAF in 1-mb sliding windows (not counting assembly gaps), where adjacent windows overlapped by 500 kb, and defined LOH blocks as genomic intervals in which the mean MAF in a given sample was less than 0.25.

### Databases annotation

Single nucleotide variants and indels were annotated using Annovar [30] (version 2011 Nov20) to classify variants as synonymous, non-synonymous, missense, or frameshift point mutations and frameshift indels. Mutations were then annotated using the COSMIC database [31] and the cancer gene census database by intersecting regions using BEDtools (v2.14.2) [25]. A Perl script was developed to run all of the annotation steps automatically and pool annotation results into one final file.

### Comparative analyses

Set theory operations (that is, union, intersect) were performed using custom perl scripts to parse the VCF4 files generated by the GATK Unified Genotyper. To build Venn Diagrams, the following filtering criteria were used: for somatic SNVs we required a minimum of 20X coverage depth with ≥5 variant reads from all tumor and normal samples, for indels we used coverage depth cutoff of 20 and variant reads of five for tumor samples. For copy number alterations we used mean KS segments with ratio ≥1.4 and ≤0.8, and for structural variants we used sites that were validated by *de novo* assembly and confirmed to be absent from the D lines by alignment-based genotyping (see SV section above). Venn diagram plots were constructed using a R package named Vennerable [32].

### Circos plots

Circular genomic visualization plots were constructed using Circos [33] (V 0.55) using BED files generated from variant calling and Annovar annotation. SNVs, indels, LOHs, SVs altering copy number were plotted as highlights. CNA were plotted as histograms. Balanced SVs were plotted as ribbons.

### Calculation of neighbor-joining trees

Neighbor-joining trees [34] were constructed using Matlab (Mathworks) by calculating distance matrices using either Euclidean distance (for copy number data) or hamming distances (for point mutations, indels, and structural variants). For Hamming distance matrices we calculated binary vectors of mutations by denoting the presence (1) or absence (0) of variants in the VCF4 files generated by GATK. All neighbor-joining trees were finally re-rooted by the diploid node in Matlab (Mathworks).

### TCGA data comparisons

Genes with non-synonymous point mutations that showed significant POLYPHEN (>0.5) or SIFT scores (>0.1) were compared to the ‘Breast Invasive Carcinoma, TCGA 2012’ and ‘TCGA provisional’ databases to determine the frequencies in patient cohorts. The cBio portal for Cancer Genomics (Memorial-Sloan Kettering) tool was used to compare the mutation sets to these databases [35].

### Pathway and network analysis

Pathways analysis was performed using the KEGG database (Kyoto) and DAVID (v6.7, NIH) analysis tool. GenBank identifiers associated with nonsynonymous mutations in genes that had significant POLYPHEN (>0.5) or SIFT scores (>0.1) were used for analysis. Pathways with significant enrichment scores (*P* >0.01) over the background frequency, normalized by the total number genes, were reported. Network analysis was performed using Ingenuity Pathway Analysis (Qiagen, Inc.). Multiple networks were identified using the non-synonymous point mutation data from all of the subpopulations, by calculating scores and identifying the networks with the highest scores. Briefly, the network score is based on the hypergeometric distribution and is calculated with the right-tailed Fisher’s exact test. The score takes into account the number of network molecules in the network and its size, as well as the total number of network eligible molecules analyzed and the total number of molecules in the Ingenuity Knowledge Base that could potentially be included in networks. The network data was plotted using Cytoscape [36].

### Computational inference of tumor subpopulations

SciClone [37] and PurBayes [38] were applied to mixed BAM files at varying coverage depths (30X, 50X, 100X) to estimate clonal subpopulations using the mutation frequency data and copy number profiles. SciClone is an R package that was downloaded as a compressed file from GitHub [39]. The run.R script was executed using the VCF4 somatic mutation variant file and the segmented copy number states to perform clustering and density estimations. The plot.R function was used to plot the allele frequency distributions and density estimations. Default parameters were used for running the scripts, except for the minimum depth parameter which was set to 50, 20 and 15 for the 100X, 50X, and 30X mixed BAM files. For the subpopulations, T10AA, T10AB, and T10H, the minimum depth was set to 20. We used PurBayes v1.3 to estimate the number of sub-clonal populations from the mixed population data. Using SNVs estimated from mixed data with coverage of 100x, 50x, and 30x respectively, we filtered SNPs in dbSNP, and selected only SNVs that were present in copy neutral regions (Logratio between −0.5 and 0.5, or −1 and 1, or −2 and 2). We further reduced the list of SNVs to only the SNVs present in the exonic regions (Exons defined by Gencode Manual Ver. 3 downloaded from the UCSC Genome Browser) and having minimum read coverage of 30 (ref + alternate). These SNVs were then analyzed with the PurBayes R package using default parameters (M = NULL, Z = NULL, pop.max = 5, prior = NULL, burn.in = 50000, n.post = 10000, fn.jags = ‘PB.jags’, plot = TRUE).

## Results

We developed a novel approach called Ploidy-Seq to study genomic diversity and mutational evolution in human cancers. The principle of this method is to use flow-sorting to purify and enrich subpopulations from polyploid tumors prior to next-generation sequencing analysis. Our cytometric data (Additional file 1: Figure S1) and previous studies [4] suggest that approximately half of all breast tumors show multiple distributions of ploidy, as do many other solid cancers types [40]. These data suggest that Ploidy-Seq will have broad applications for studying genome evolution in many human cancers. After enrichment and isolation, we perform whole-genome deep-sequencing of the tumor subpopulations to detect the full spectrum of somatic mutations, including point mutations, indels, CNAs, structural variants, and loss of heterozygosity (LOH). From these data we apply set theory operations and lineage analysis to infer the relative chronology of mutations that occurred during tumor evolution.

**Figure 1:**
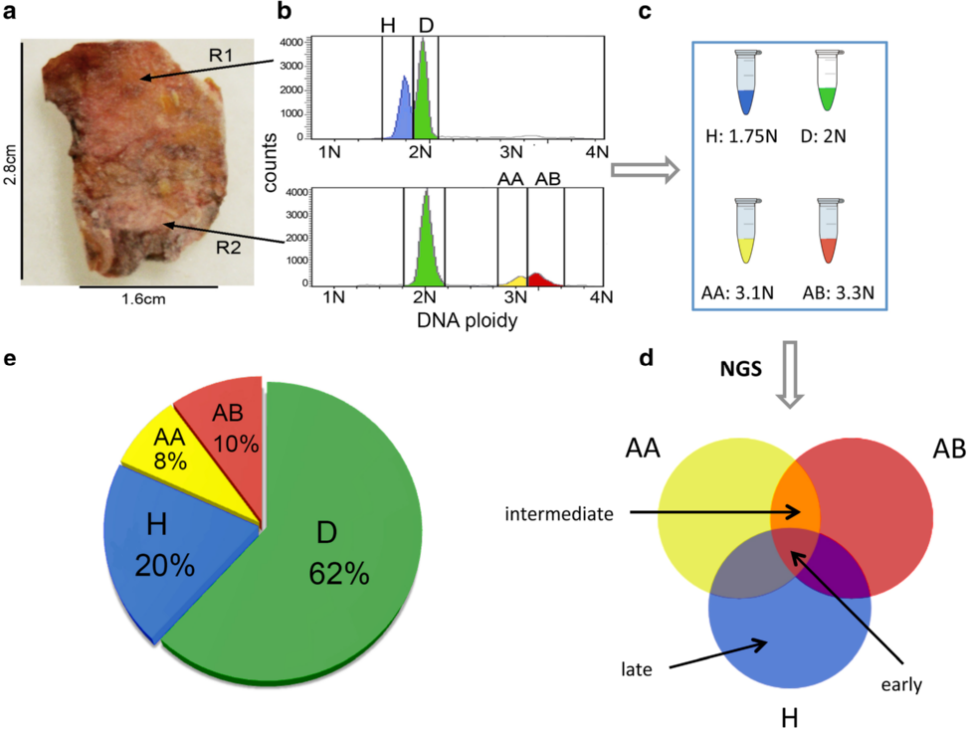
**Ploidy-Seq approach.** Outline of the approach for isolating and enriching tumor subpopulation by differences in total DNA content prior to next-generation sequencing. Images and cytometric data are displayed from the triple-negative breast tumor case. **(a)** Two regions (R1 and R2) were sampled and nuclear suspensions were prepared and stained with DAPI. **(b)** The nuclei were flow-sorted and different distributions of ploidy were gated. **(c)** Cells from each ploidy peak were isolated into separate tubes and the DNA was isolated and used to prepare libraries for next-generation sequencing. **(d)** Somatic mutations from each subpopulation were detected and set theory operations were performed to identify early intermediate or late mutations. **(e)** The composition of the tumor subpopulations relative to the total mass were calculated from the number of events in the cytometric data.

We applied Ploidy-Seq to study an invasive ductal carcinoma from a 53-year-old triple-negative (ER-/PR-/Her2-) breast cancer patient. Histopathology revealed a high-grade (III) invasive ductal carcinoma with significant immunocyte infiltration. We flow-sorted nuclei from two regions (R1 and R2) in the frozen tumor sample (Figure 1a). The upper region showed a diploid distribution (2 N) and a hypodiploid (H) distribution (1.7 N). The lower regions showed a diploid distribution (2 N) and two aneuploid distributions at 3.1 N (AA) and 3.3 N (AB). From the cell count cytometric data we estimated the total proportions of cells in the bulk tumor (Figure 1b). We collected millions of nuclei from each region and ploidy peak and performed whole-genome deep-sequencing on the four subpopulations (D, H, AA, and AB) at 53X mean coverage on the Illumina HiSeq2000 platform using 100 bp paired-end reads (see Methods).

The sequence reads were aligned to the human genome using BWA and variants were detected using a comprehensive data processing pipeline (Methods and Additional file 1: Figure S2). Point mutations and indels were detected using GATK [21], copy number profiles were detected using varbins [12], and structural variants (SVs) were detected using CREST [26] and a custom split-read mapping pipeline (methods). Somatic mutations were distinguished from germline variants based upon their presence in one or more tumor subpopulations (H, AA, and AB) but not the diploid subpopulation (D), which derive from normal infiltrating cells. After removing germline variants and applying various filters (methods), we detected a total of 17,630 single nucleotide variants (SNVs), 4,510 indels, 657 structural variants (SVs), and 312 copy number alterations (CNAs). In the coding regions we detected 83 non-synonymous SNVs, four frameshift indels, and two SVs (Additional file 2: Tables S1-S3).

### Identifying unique and common somatic mutations

**Figure 2:**
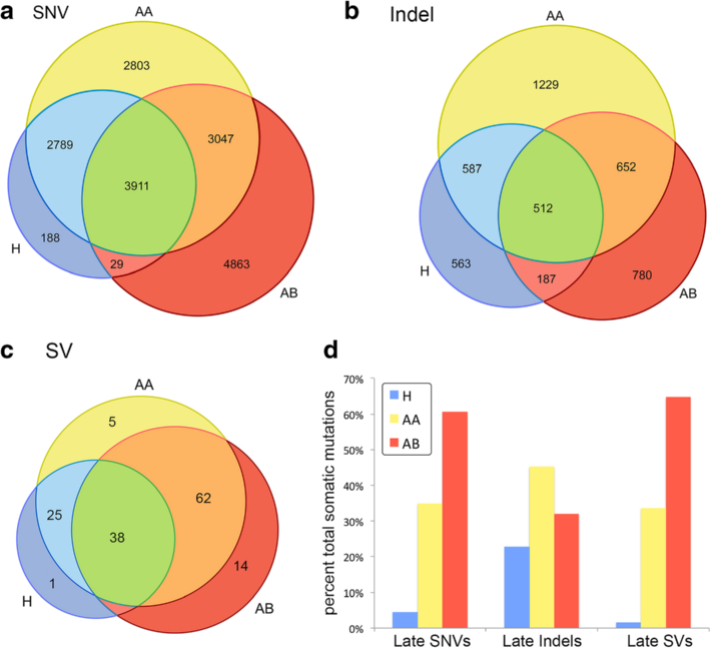
**Venn diagrams of shared and unique somatic mutations.** Venn diagrams were constructed from different classes of somatic mutations by comparing the three tumor subpopulations (H, AA, and AB). **(a)** Single nucleotide variants, **(b)** small insertions and deletions, **(c)** structural variants, and **(d)** barchart of the somatic mutation percentages in each tumor subpopulation.

We applied set theory operations to classify the somatic mutations as: early (present in all subpopulations), intermediate (shared between two subpopulations) or late (exclusive to one subpopulation) (Figure 2a-c). Strikingly, we found that only 22.18% of the point mutations and 8.18% of the indels were shared between all three tumor subpopulations. The majority of the somatic mutations were present in two subpopulations or exclusively detected in only one subpopulation. The AB subpopulation harbored the highest number of private point mutations (N = 4863) and structural variants (N = 81) compared to the other subpopulations. In contrast the AA subpopulation showed the highest number of private indels (N = 1,012). We plotted the percentage of mutation classes by subpopulation, which clearly shows that the H subpopulation showed the fewest numbers of mutations for all classes, suggesting that it represents one of the earliest subpopulation in the tumor (Figure 2d). We also examined the mutation spectrum of transitions and transversions, which showed no clear differences between the subpopulations (KS test, *P* = 0.34). In all three subpopulations we observed only marginal (5%) increases in C > T (G > A) transitions (Additional file 1: Figure S4). These mutation spectrums are consistent with recent reports from other breast cancer genome sequencing studies [41-43].

### Inferring the evolutionary lineage of the tumor subpopulations

**Figure 3:**
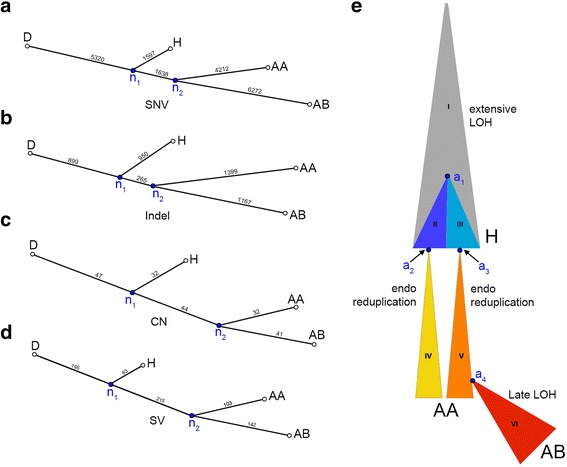
**Genomic lineage of tumor subpopulations. (a-d)** To visualize the genetic distance between tumor subpopulations, neighbor-joining trees were constructed from distance matrices using different classes of somatic mutations. **(a)** Single nucleotide variant tree. **(b)** Indel tree. **(c)** Copy number aberration tree. **(d)** Structural variant tree. **(e)** Diagram of one potential evolutionary history that can explain the patterns of variant sharing shown in Figure 2a. Different clonal lineages inferred by Ploidy-Seq are indicated by distinct colors and roman numerals. Note that the relative area of each clone is not representative of the actual abundance in the analyzed tumor. Key cellular ancestors are indicated by the labels a1-a4: a1 is the last common ancestor of the genetically distinct hypodiploid clones II and III; a2 and a3 are the cells in which endoreduplication occurred to produce clone IV and clone V, which comprise the AA population; a4 is the cell giving rise to AB (clone VI), which is the most highly mutated subpopulation and appears to have undergone rapid clonal expansion.

To gain insight into the evolutionary relationship among tumor cell subpopulations, we constructed neighbor-joining trees using different classes of somatic mutations (SNV, indel, SV, and CNA). We found that all trees shared a common topology, in which the hypodiploid (H) subpopulation was ancestral to the two sub-tetraploid AA and AB subpopulations (Figure 3a-d). A simple interpretation of this data is a linear model of clonal evolution whereby a diploid progenitor lineage underwent catastrophic loss of whole chromosomes and chromosome arms to form an approximately 1.7 N hypodiploid cell population, followed by a single genome doubling event that gave rise to the two sub-tetraploid subpopulations AA (3.1 N) and AB (3.3 N).

However, this naive linear model is based purely on genetic distance, and is contradicted by the large number of somatic mutations that are shared between the AA and H subpopulations, but absent from AB (for example, 2789 AA-H SNVs, Figure 2a). If the AA and AB subpopulations derived from the same genome doubling event, which necessarily must have occurred in a single cell, then due to this single cell ‘bottleneck’ event they should both retain all of the early somatic mutations that were shared between that single cell and the H subpopulation as a whole, yet they clearly do not. One possible explanation is that the 2,789 AA-H SNVs are early mutations that were lost from the AB lineage due to late LOH events, a plausible hypothesis given that AB has many private LOH blocks (Additional file 1: Figure S5). To test this hypthesis, we examined overlap between SNVs and LOH events, and found that only 257 of the 2,789 (9.2%) AA-H SNVs reside in AB-specific LOH blocks, which is merely a 2.04-fold enrichment relative to the number expected by chance (Additional file 1: Table S4). Therefore, the vast majority of AA-H SNVs cannot be explained by LOH events.

This discrepancy strongly argues that multiple genome doubling events occurred during the evolution of the AA and AB subpopulations. One model to account for these observations is that two endoreduplication events occurred in genetically distinct clones of the hypodiploid ancestral population, at approximately the same time and physical location during tumor evolution, leading to a sub-tetraploid AA cell population composed of two intermixed lineages (Figure 3e). The AB subpopulation then arose from a small number of cells derived exclusively from one of the two clones in the AA subpopulation, and subsequently underwent rapid expansion. This recent bottleneck and clonal expansion is supported by the high allele frequency of SNPs contained within LOH events in the AB relative to the AA and H subpopulation (Additional file 1: Figure S5 and Figure S6a), as well as the relatively high frequency of AB-specific SNVs (Additional file 1: Figure S6f).

An alternative model invokes cell fusion (Additional file 1: Figure S7b). Here, a hypodiploid ancestor of AA and AB diverged from the H subpopulation for a long period of time, acquiring numerous mutations that are not present in H (AA-AB markers). This ancestral lineage gave rise to the AA sub-tetraploid clone through a cell fusion event with a cell closely related to the H subpopulation, and gave rise to the AB sub-tetraploid clone through an endoreduplication event. Although cell fusion has been studied extensively in cell culture and has been proposed as a mechanism of tumor evolution [44], it has to our knowledge not been definitively shown to occur in endogenous human tumors (although some anecdotal evidence has been reported [45]). Thus, we conclude that the first model is more likely, since it involves two independent occurrences of endoreduplication, which is well established mechanism for tetraploidization [46]. Moreover, although most of the observed SNV allele frequency distributions (Additional file 1: Figure S6) are consistent with either model, the cell fusion model strongly predicts that AA-AB SNVs should be fixed within both the AA and AB lineages due to their respective single cell bottleneck events, and therefore be present at an allele frequency of precisely 25% in the AA subpopulation (one of four chromosomes due to cell fusion) and 50% in AB (two of four chromosomes due to endoreduplication). In contrast, the observed mean allele frequencies of AA-AB SNVs in AA and AB subpopulations are substantially lower (13% and 32%, respectively), which is more consistent with the dual endoreduplication model.

Thus, our data strongly suggest that the AA subpopulation is composed of two major clones, each derived from an independent endoreduplication event in genetically distinct cells of the ancestral H subpopulation. This is to our knowledge the first evidence that multiple genome doubling events may occur within a single tumor, and suggests that such events may be surprisingly common. A caveat is that these data also show an inherent limitation of Ploidy-Seq: it is not possible by flow sorting to distinguish between cells that have distinct evolutionary histories yet similar ploidy, and thus it is important to recognize that genetically distinct clones may exist within flow-sorted cell populations. Nonetheless, by greatly simplifying the problem of reconstructing tumor evolution, Ploidy-Seq allows novel insights that would be missed by bulk sequencing.

### Inferring the ancestral tumor genome

**Figure 4:**
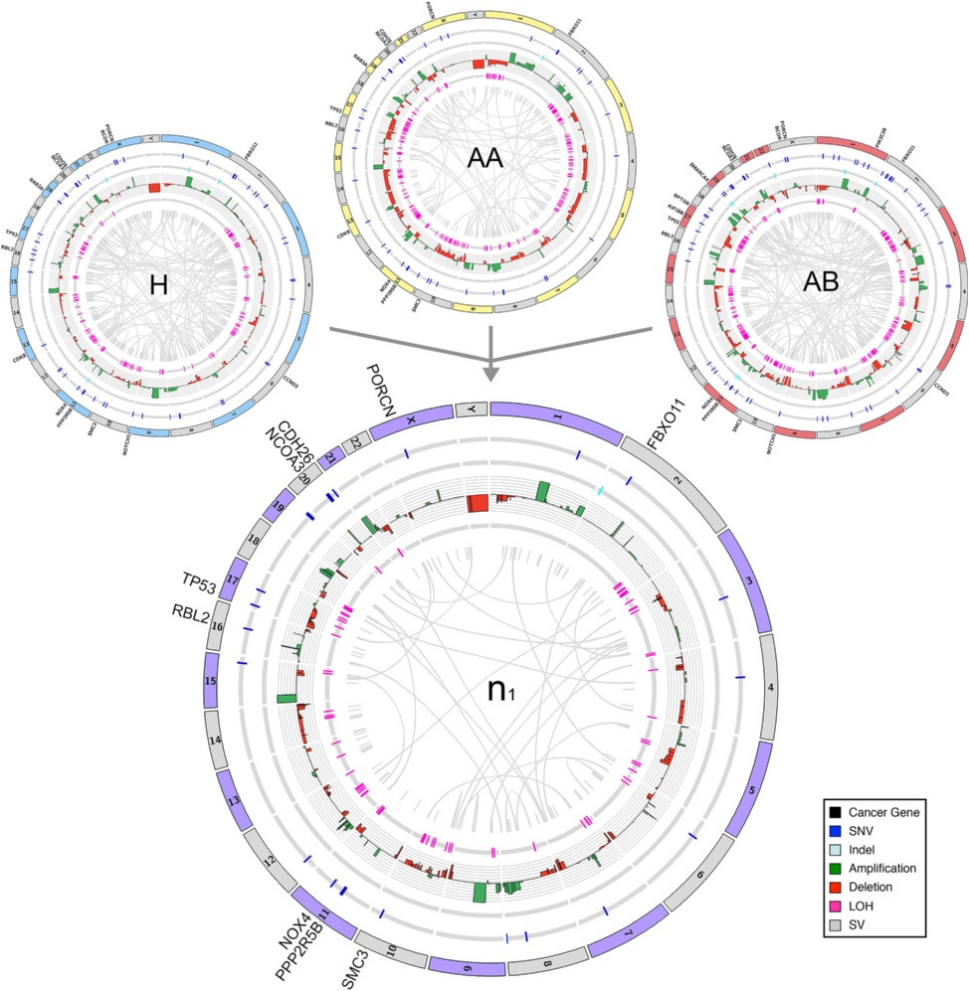
**Inferred ancestral tumor genome.** Circos plot of the n1 ancestral tumor genome that was inferred by identifying the common set of somatic mutations in the H, AA, and AB tumor subpopulations. Somatic mutations in cancer genes are displayed on the outer ring, while different classes of somatic mutations are displayed in the inner rings.

We used the mutational data from the three tumor subpopulations to infer the ancestral genome (n_1_) from which all subpopulations emerged (Figure 4). This ancestral genome showed a reduced number of somatic mutations (SNVs, indels, LOH, SVs, and CNAs) compared to the advanced subpopulations, allowing us to identify mutations that played an important role in the early stages of tumor progression. Among the 83 non-synonymous mutations that occurred throughout tumor evolution, we identified nine early non-synonymous point mutations that occurred in cancer genes (Additional file 2: Table S1). Only five of these mutations (*TP53*, *PPP2R5B*, *FBXO11*, *PORCN*, and *NOX4*) were predicted to have damaging effects on protein function as predicted by SIFT (<0.1) and POLYPHEN (>0.5) scores [47,48]*.* Among these, we found a salient mutation in exon 7 in the DNA binding domain of *TP53*. This mutation (Y234C) was previously reported 82 times in the COSMIC database [31] at the same nucleotide position, suggesting that it is a strong driver mutation. However, the *TP53* mutation was unlikely to be acting alone: two other early mutations (*PPP2R5B* and *FBX011*) were detected, and have previously been reported to interact with *TP53* to promote tumor growth. *PPP2R5B* was reported to have a tumor suppressor activity by interacting with *PP2A* to mediate dephosphorylation of *TP53* in response to DNA damage [49]. *FBX011* is an adapter protein that has been shown to promote neddylation of p53 and inhibit its transcriptional activity [50]. We also detected an early mutation in *PORCN,* which is an upstream regulator of the *WNT* signaling pathway and in *NOX4*, which is an NADPH oxidase involved in the generation of hydrogen peroxide and superoxide [51]. We speculate that the *NOX4* mutation may have increased the reactive oxygen species and mutation rate in the tumor microenvironment. We also detected an early structural rearrangement, a 1,058 bp deletion in exon5 of *MLLT3*, a gene that is often translocated in leukemias (Additional file 2: Table S3), and early copy number changes, including a gain of chromosome 1q and loss of 1p, amplification of chromosome 8q (*MYC*) and loss of 17p. These mutations may have acted together to drive the early stages of tumor progression in this patient.

### Inferring the chronology of somatic mutations

**Figure 5:**
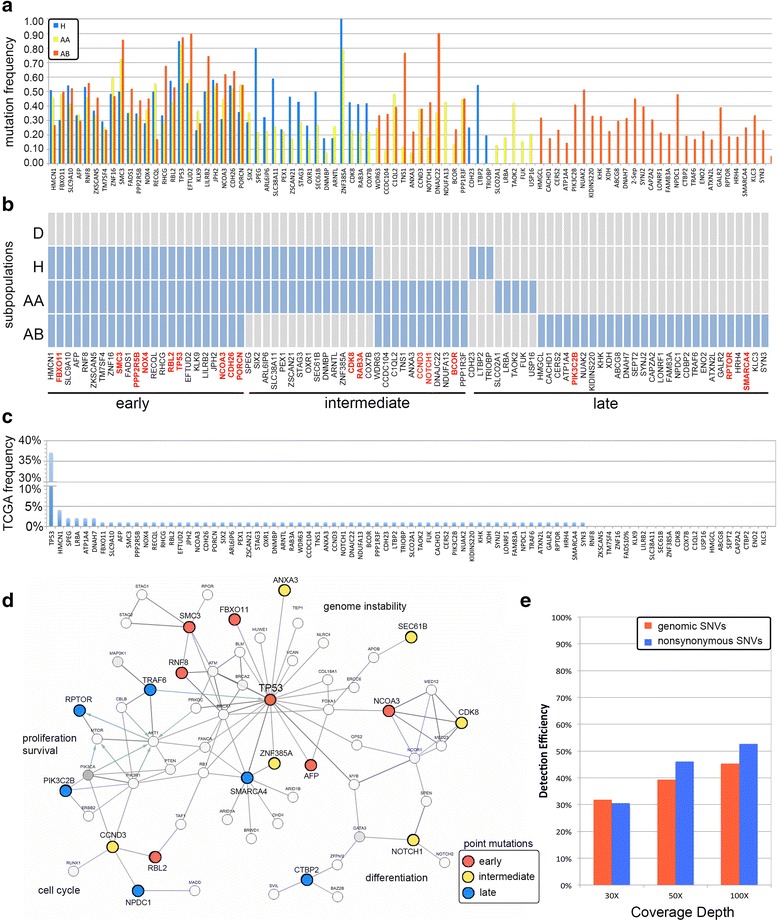
**Chronology of somatic mutations and interaction networks. (a-c)** Non-synonymous point mutations detected in the breast tumor subpopulations **(a)** Distribution of allele frequencies of the non-synonymous mutations. **(b)** Heatmap of the non-synonymous mutations ordered by relative chronology. Point mutations in cancer genes are highlighted in red. **(c)** Percentage of somatic mutations detected in the TCGA breast tumor patient cohort. **(d)** Network analysis of the point mutations that were temporally deregulated during tumor evolution. **(e)** Detection efficiency of simulated bulk data at different coverage depths.

We also identified a number of mutations that occurred during the intermediate and late stages of tumor progression (Figure 5) (Additional file 2: Table S1). Overall, we observed that early mutations showed higher allele frequencies when compared to the intermediate (*P* = 0.026, *t*-test) and late (*P* = 0.0015, *t*-test) mutations (Figure 5a). As expected from the phylogenetic analysis, many of the intermediate nonsynonymous mutations were shared between the H and AA subpopulations, and the AA and AB subpopulations, but not shared between H and AB. Two intermediate mutations in the H and AA subpopulations (*CDK8* and *RAB3A*) were predicted to have damaging effects on protein function by SIFT and POLYPHEN scores (Additional file 2: Table S1). These mutations were not detected in the AB subpopulation, suggesting that they are not likely to be important in the late stages of tumor progression. After the divergence of the second common ancestor (n_2_) we detected mutations in *NOTCH1*, *BCOR*, and *CCND3*, which all showed significant POLYPHEN and SIFT scores (Additional file 2: Table S1). The *NOTCH1* mutation (M1615I) occurred in the NOD domain and is likely to be a driver mutation, since it was previously reported at the same nucleotide position in the COSMIC database. *NOTCH1* is involved in many cellular signaling processes associated with development and cell fate and has recently been suggested to be a novel oncogene in breast cancer [52].

Interestingly, many of the late mutations occurred exclusively in the minor AB subpopulation. We detected three non-synonymous mutations in cancer genes, including a nonsense mutation in *RPTOR* and point mutations in *PIK3C2B* and *SMARCA4. RPTOR* is a gene that interacts with mTOR to regulate the signaling of cellular proliferation and survival [53]. *PIK3C2B* is a member of the PI3 Kinase family pathway involved in regulating cell proliferation, survival, and migration [54]. The AB subpopulation also showed a 50-fold copy number amplification of the *KRAS* locus on chromosome 12p12.1 and two homozygous deletions of the *EFNA5* and *COL5A4* tumor suppressors. Thus, while AB was the rarest subpopulation in the tumor (about 8% of the tumor mass) it also harbored the largest number of cancer gene mutations. Interestingly, the AB allele frequencies suggest that AB underwent a recent population bottleneck (Additional file 1: Figure S5f), and thus we infer that these mutations provided a strong selective advantage, allowing the subpopulation to expand rapidly.

We compared all of the significant somatic mutations detected in this study to the breast cancer mutations reported in the Cancer Genome Atlas (TCGA) (Figure 5c). We found that the majority (74%) of the point mutations were previously reported in TCGA, but often occurred at low frequencies in the patient cohort (<3%). The exception was *TP53*, which was reported to occur in 37% of the TCGA breast cancer patients.

### Pathway and network analysis

To determine if any signaling pathways were deregulated by acquiring multiple somatic mutations during tumor evolution, we used the non-synonymous point mutations to perform pathway analysis (methods). Our data suggest that the *Wnt* Signaling pathway was the only pathway to show a statistically significant enrichment (*P* = 0.083) of mutations. Interestingly, mutations in this pathway accumulated progressively as the tumor evolved. The earliest mutations occurred in *PORCN*, *CDH26*, *PPP2R5B*, and *TP53* and were present in all three subpopulations (H, AA, AB). This was followed by an intermediate mutation in *CCND*3 that occurred in the n_2_ ancestor and was present in both AA and AB. Finally late mutations occurred in *CTBP2* and *SMARCA4* in the AB subpopulation. Wnt signaling is involved in regulating cell-to-cell interactions during embryogenesis, and many mutations in this pathway have been associated with cancer progression [55,56].

We also performed network analysis to identify larger networks beyond individual pathways that were disrupted during tumor evolution (methods). These networks are based on direct protein interaction data or protein modifications (Figure 5d). Consistent with our previous results, the network analysis revealed multiple mutations involved in regulating genome instability in the *TP53* network, including *TP53*, *FBXO11*, *PPP2R5B*, *RBL2*, and *SMC3* (*P* = 0.009, Fisher’s exact test). The extended network also included early mutations involved in regulating nuclear transcription (*NCOA3*) and cell cycle (*RBL2*). We also found an intermediate mutation in cyclin D3 (*CCND3*), which interacts with *RBL2*, and a mutation in *NOTCH1* involved in regulating differentiation. The late mutations in this network were found to be involved in regulating cell proliferation. A late mutation was found in *PIK3C2B* which interacts with *PIK3CA*, one of the most frequent mutations that has been reported in breast cancer [43,57]. The other late mutation in this network occurred in *RPTOR*, a gene that regulates cell proliferation and apoptosis. All of the late mutations that deregulated the TP53 network (Figure 5d) occurred exclusively in the AB subpopulation, which comprised the minority of the tumor mass. The early mutations occurred in all three subpopulations, while the intermediate mutations were found in both the AA and AB subpopulations. In summary, these data shows a complex interplay of different cellular processes (genome instability, cell cycle, proliferation, survival, apoptosis, and differentiation) that were deregulated at progressive stages of tumor growth.

### Simulating data from bulk tumor tissues

In order to estimate the number of mutations that would have been detected by sequencing the bulk tumor *en masse*, we performed *in silico* mixing experiments. We combined raw sequence data from the three tumor subpopulations in proportion to the numbers of cells detected in the cytometric data to obtain mixtures of reads corresponding to 30X, 50X, and 100X mean sequence coverage (see Methods). From these data we detected genome-wide somatic SNVs using the same processing pipeline that was used to detect mutations in the tumor subpopulations (methods). We then compared the total number of point mutations detected from deep-sequencing the three tumor subpopulations separately to the combined simulation data at different sequencing depths. We found that only 30% of the mutations were detected at 30X coverage, while 38% were detected at 50X and 43% were detected at 100X (Figure 5e). Thus, our data suggest that the majority of mutations would have been missed entirely by sequencing the bulk tumor *en masse* at standard sequencing depths.

### Computational estimations from bulk sequencing data

Several recent computational methods have been developed to estimate clonal subpopulations from bulk NGS data by clustering mutation frequencies [37,58-60]. We applied two computational methods (SciClone and PurBayes) that cluster mutation frequencies and use Bayesian mixture models to identify the correct number of clusters that correspond to clonal subpopulations. In these methods, it is assumed that the mutation clusters correspond directly to the individual subpopulations in the tumor [30,31]. To perform these analyses we used the *in silico* mixed datasets to represent bulk sequencing at different coverage depths (30X, 50X, and 100X). Both methods used the copy number profiles of the mixed data to identify copy-neutral mutations, which were used filter regions of the genome that contain CNAs that might skew the mutation frequencies. SciClone analysis [37] predicted only a single cluster that corresponded to one subpopulation of tumor cells, even at the highest (100X) coverage depth (Additional file 1: Figure S8). Similarly, PurBayes [38] identified only a single cluster that corresponded to one clonal subpopulations at 30X or 50X coverage depth (Additional file 1: Figure S9a, b). However, PurBayes did stratify the 100X mixed SNV dataset into two separate clusters that may potentially correspond to clonal subpopulations. However, when we compared the mutations from these two clusters to the H, AA, and AB subpopulations detected by Ploidy-Seq, we found no clear correlation: the mutations in each cluster were found in all three subpopulations. Next, we sought to determine if the computational methods could reveal further substructure in the flow-sorted subpopulation datasets (H, AA, and AB). We applied SciClone to the data generated by Ploidy-Seq and found that no further clusters or subpopulations could be detected in the H, AA or AB datasets (Additional file 1: Figure S8b-d). Therefore, we conclude that bulk tumor sequencing and computational inference methods could not resolve the same population substructure that was resolved by Ploidy-Seq analysis in this tumor.

## Discussion

In this study we present a novel approach for reconstructing genome evolution from polyploidy tumors. Our method involves isolating and comparing somatic mutations in different subpopulations to trace lineage and infer mutational chronology. We applied this method to study genome evolution in a triple-negative breast tumor, by sequencing three tumor subpopulations and a population of diploid cells. Our data revealed a highly complex tumor, harboring thousands of somatic mutations, including point mutations, indels, structural variants, and copy number aberrations. Strikingly, we found that only 22.18% of the point mutations and 8.18% the indels were shared between all three subpopulations. These results are consistent with a recent report in which tumor cells were spatially sampled from kidney tumors [61] and found to share only 63% to 69% of SNVs among all sampled regions. The shared mutations we identified were present in all of the tumor subpopulations and thus represent the earliest mutations that occurred during tumor progression. Among them we identified an early driver mutation in exon 7 of *TP53*. There is much debate in the field as to whether *TP53* plays an important role in the early stages of tumor progression [62]. Our data strongly suggest that the *TP53* mutation was an early founder mutation that was inherited by all subsequent subpopulations. However, the *TP53* mutation was unlikely to be operating alone, since we also detected mutations in *FBXO11* and *PPP2R5B,* which have been shown to interact with *TP53* to drive tumor growth [49,50]. These early mutations were present in all of the tumor cells, suggesting origin from a common ancestor that evolved from a single cell in the normal breast tissue of this patient.

Using genetic mutations as stable markers of evolution, we reconstructed the phylogenetic lineage of the tumor. A simple interpretation of the data was a linear pathway in which a single normal cell underwent genome-wide chromosome losses to form the H subpopulation (1.7 N) followed by a genome duplication to generate the AA (3.1 N) and AB (3.3 N) subpopulations. This model is supported by neighbor joining trees constructed from multiple mutation datasets (SNVs, indels, CNAS, and SVs). However, this model is contradicted by a large number of somatic mutations that are shared between the H and AA subpopulations, but absent from AB, which cannot be explained by trivial reasons such as LOH. Instead, these mutations imply a more complex model of evolution in which two independent genome duplication events occurred in the lineage giving rise to the AA and AB subpopulations. The mechanism underlying these genome duplication events are unknown, but may have been due to either cell fusion or endoreduplication. Our current data are not sufficient to definitively distinguish between these mechanisms, but the observed SNV allele frequencies (Additional file 1: Figure S6) are more consistent with endoreduplication. Endoreduplication is a well established mechanism for genome duplication in human cancers, while evidence for cell fusion has been limited to cell culture experiments [44] and a single tumor from a bone marrow transplant patient [45].

The genome doubling events subsequently led to the formation of two minor subpopulations (AA and AB) in the tumor that comprised approximately 8% and 10% of the total tumor mass. While minor, these subpopulations contained many additional somatic mutations, including shared mutations in cancer genes including *NOTCH1*, *CCND3*, and *BCOR*. The AB subpopulation showed the highest number of somatic mutations, including point mutations in *RPTOR* and *PIK3C2B* and a massive 50-fold amplification in the *KRAS* oncogene as well as focal homozygous deletions of the *EFNA5* and *COL4A5* tumor suppressors. These mutations may have led to an increase in cell proliferation and migration, and an inhibition of apoptosis. Our data suggest that the minor subpopulations were the most malignant cells in the tumor. Importantly, this was a treatment-naïve tumor sample, and thus the evolutionary lineages occurred through natural selective pressures in the tumor microenvironment, rather than selective agents.

Recent reports from large-scale sequencing projects have identified thousands of nonsynonymous mutations in breast cancer genomes [7,15,43,57], but surprisingly few mutations that are shared between individual patients. In triple-negative breast cancer *TP53* is the only mutations that occurs at a high percent of patients (80%), while the vast majority of mutations (including *PIK3CA*, *RB1*, *PTEN*, *MYO3A*, and *GH1*) occur at low percentages (<10%) in the patient cohorts [7]. On average, these studies identified only a few (one to five) driver mutations in cancer genes in each patient’s tumor. In contrast, we identified 20 non-synonymous driver mutations in cancer genes in this TNBC patient, many of which occurred in the minor subpopulations. When we compared these mutations to the TCGA data, we found that most of the mutations (74%) were previously detected in other breast cancer patient genomes. Thus, our data may indicate that many additional somatic mutations may be shared between different patients, but reside in minor tumor subpopulations and thus go undetected at standard sequencing depths. We simulated reads at different sequence depths for variant detection and found that most of the late and intermediate mutations would have been missed entirely by bulk sequencing at standard coverage depth. These data show the importance of using methods such as Ploidy-Seq or ultra-deep sequencing in order to identify mutations in minor subpopulations that may play an important role in tumor progression, invasion, and metastasis.

We previously analyzed copy number evolution in this tumor using single-cell sequencing [11] and spatial sampling by microarray CGH [4]. The copy number profiles from these studies are consistent with our current analysis, by showing that the tumor cells were organized into three major clonal subpopulations (H, AA, and AB), each sharing the majority of amplification and deletions. These data also showed a common genetic lineage, suggesting an origin from a single somatic cell in the breast. However, the current studies provide further insight into the clonal substructure and evolution of point mutations, indels, and structural variants in addition to CNAs that occurred during tumor evolution.

Our data have important implications for the clinical diagnosis and treatment of TNBC patients. First, we show that tumor subpopulations can be spatially segregated in the tumor and carry distinct sets of somatic mutations that reside in different geographical regions. This has important implications for diagnostic testing, because we would not have detected mutations in *KRAS* or *RPTOR* in the upper regions of the tumor mass. This ‘spatial heterogeneity’ in tumors is becoming more widely recognized in solid tumors [4,61,63] as research studies begin to sample multiple regions within solid tumors for genomic studies. Our data also have important implications for targeted therapy, by showing that early mutations are likely to be ideal targets for therapy, since they are molecular targets that are present in the majority of the tumor cells. In theory, we should be able to eradicate the entire tumor mass by targeting these mutations, since they are present in most of the tumor cells. Alternatively, we can design different therapeutic strategies to target each of the tumor subpopulations independently using the intermediate and late mutations.

To identify potential drug targets in this TNBC patient we annotated all cancer gene mutations using the Drug-Gene Interaction Database (Additional file 2: Table S1). We did not identify any early mutations that could serve as direct targets for therapy, since *TP53* is notoriously difficult to target in cancer cells without disrupting its function in normal cells. However, we did identify an intermediate mutation in *CDK8* that is present in the H and AA subpopulations that may be targeted with Flavopiridol, a cyclin-dependant kinase inhibitor. We also identified an intermediate mutation in Cyclin D (*CCND3*) that could be targeted with several drugs that are currently in clinical trials (LY2835219, LEE011,BAY1000394), or Palbociclib. In the late AB subpopulation we also identified mutations in *PIK3C2B* and *RPTOR*, which may sensitize the tumor to a number of drugs that target the PI3K/Akt/mTOR signaling pathways. Targeting the AB subpopulation specifically might have been very beneficial to the patient, since we suspect that the AB subpopulation was the most malignant in the tumor.

While Ploidy-Seq can provide a powerful approach for study genome evolution and clonal diversity in human tumors, there are also several notable limitations. Foremost, the method requires tumors that are polyploidy and have aneuploid peaks that do not overlap in ploidy with the diploid peaks, or with each other. We estimate that about half of all breast tumors contain such ploidy distributions based on our cytometric analysis (Additional file 2: Table S1). Another possible limitation (and all sequencing studies) is the sensitivity with which the mutations were detected in each of the tumor subpopulations. At a mean depth of 53.75X it is possible that some mutations, which we classified as ‘private’, do in fact exist in other tumor subpopulations, but at frequencies below our detection limits. These mutations may be detected at higher sequence read depths, but then become difficult to distinguish from sequencing errors without methods such as duplex sequencing [64]. In summary, the genomic diversity reported in this study is likely to be an underestimate of the clonal diversity in the tumor.

Finally, we note that the data presented here were derived from a single cancer patient, and thus our findings may not be generalizable to all TNBC patients. Future work will be needed, in a larger set of TNBC patients, to determine if: (1) tetraploidy through chromosome loss is a common mechanism of genome evolution; (2) large numbers of private mutations are common in tumor subpopulations; (3) tumor subpopulations are often geographically segregated with the tumor mass; and (4) early mutations in *TP53* drive tumor growth in breast cancer. Furthermore, it will be of great interest to identify the selective pressures in the tumor microenvironment (immune system, hypoxia, geographic isolation, nutrient deprivation) that cause lineages to diverge, resulting in the intratumor heterogeneity. We fully expect that these findings and novel methods for resolving intratumor heterogeneity will lead to improvements in the clinical diagnosis and therapeutic targeting of breast cancer patients.

## Conclusions

Ploidy-Seq provides a powerful new approach to isolate and study subpopulations within solid tumors. Using this tool, we studied genomic diversity and evolution in a triple-negative breast cancer patient, which revealed several important biological findings regarding genome evolution. Our data show that breast tumors can evolve by chromosome loss, followed by genome duplication to generate tetraploidy. We also identified a large number of private mutations that resided in minor tumor subpopulations and were not shared by the neighboring tumor cells. By inferring the ancestral tumor genome, we identified a subset of somatic mutations that play an important role in the early stages of tumor progression, including an early driver mutation in *TP53*. These data provide new insight into genome evolution in breast cancer and would not have been revealed by sequencing the bulk tumor *en masse*.

## Additional files

Additional file 1: Figure S1. Ploidy distributions of eight breast tumor samples. **Figure S2.** Data processing and variant detection pipeline. **Figure S3.** Structural variant detection and validation. **Figure S4.** Mutation spectrum frequencies in tumor subpopulations. **Figure S5.** Genome-wide LOH and SNV plots of tumor subpopulations. **Figure S6.** Tumor subpopulation allele frequency distributions. **Figure S7.** Evolutionary models of tumor progression. **Figure S8.** Inferring clonal substructure from mixed sequencing data and flow-sorted subpopulations using SciClone. **Figure S9.** Estimating tumor subpopulations from mixed sequencing data using PurBayes. Additional file 2: Table S1. Non-synonymous point mutations in tumor subpopulations. **Table S2.** Coding indels in tumor subpopulations. **Table S3.** Structural variants and copy number aberrations in genic regions. **Table S4.** LOH and genotype intersection table.

## Abbreviations

BWA: Burrow-Wheeler Alignment
CNA: Copy number alteration
GATK: Genome Analysis Toolkit
Indel: Insertion-deletion
NGS: Next-generation sequencing
SNV: Single nucleotide variant
SV: Structural variation
TCGA: The Cancer Genome Atlas
TNBC: Triple-negative breast cancers
