# Supplementary material for: Ploidy-Seq: inferring mutational chronology by sequencing polyploid tumor subpopulations
Source: Genome Med. 2015 Jan 28;7(1):6. doi: 10.1186/s13073-015-0127-5 (PMC4343275; doi:10.1186/s13073-015-0127-5)

**Supplemental Figure 1**

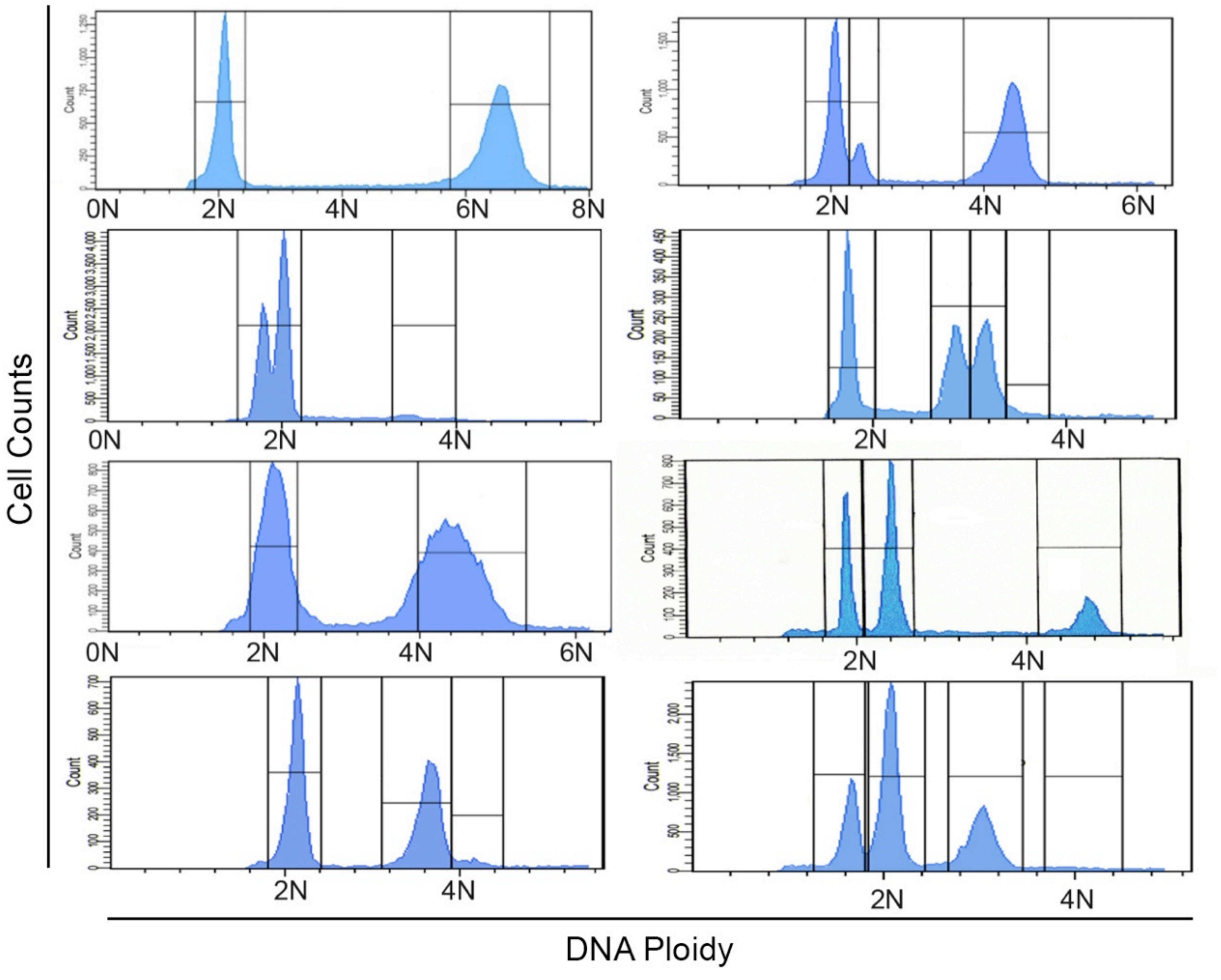

# Supplemental Figure 2

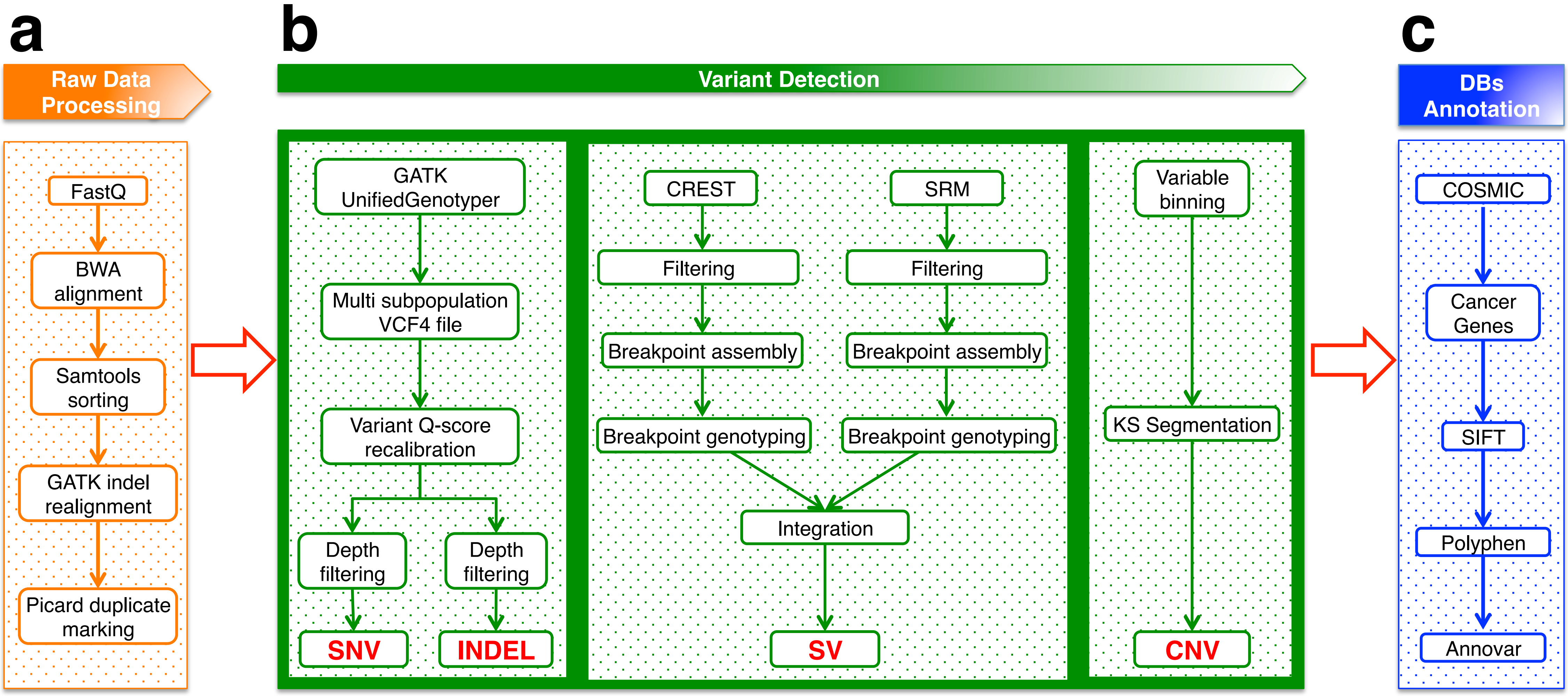

# Supplemental Figure 3

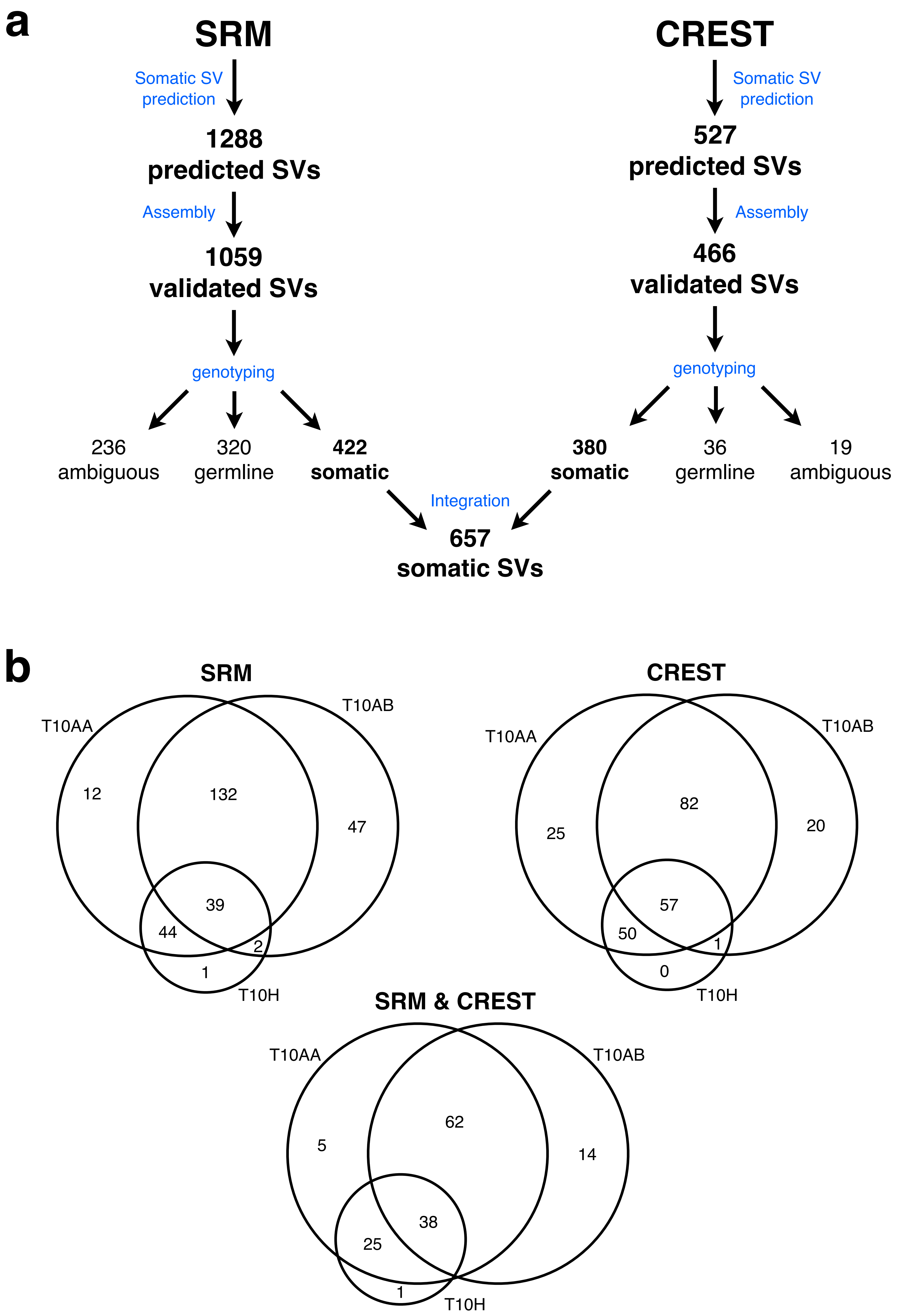

**Supplemental Figure 4**

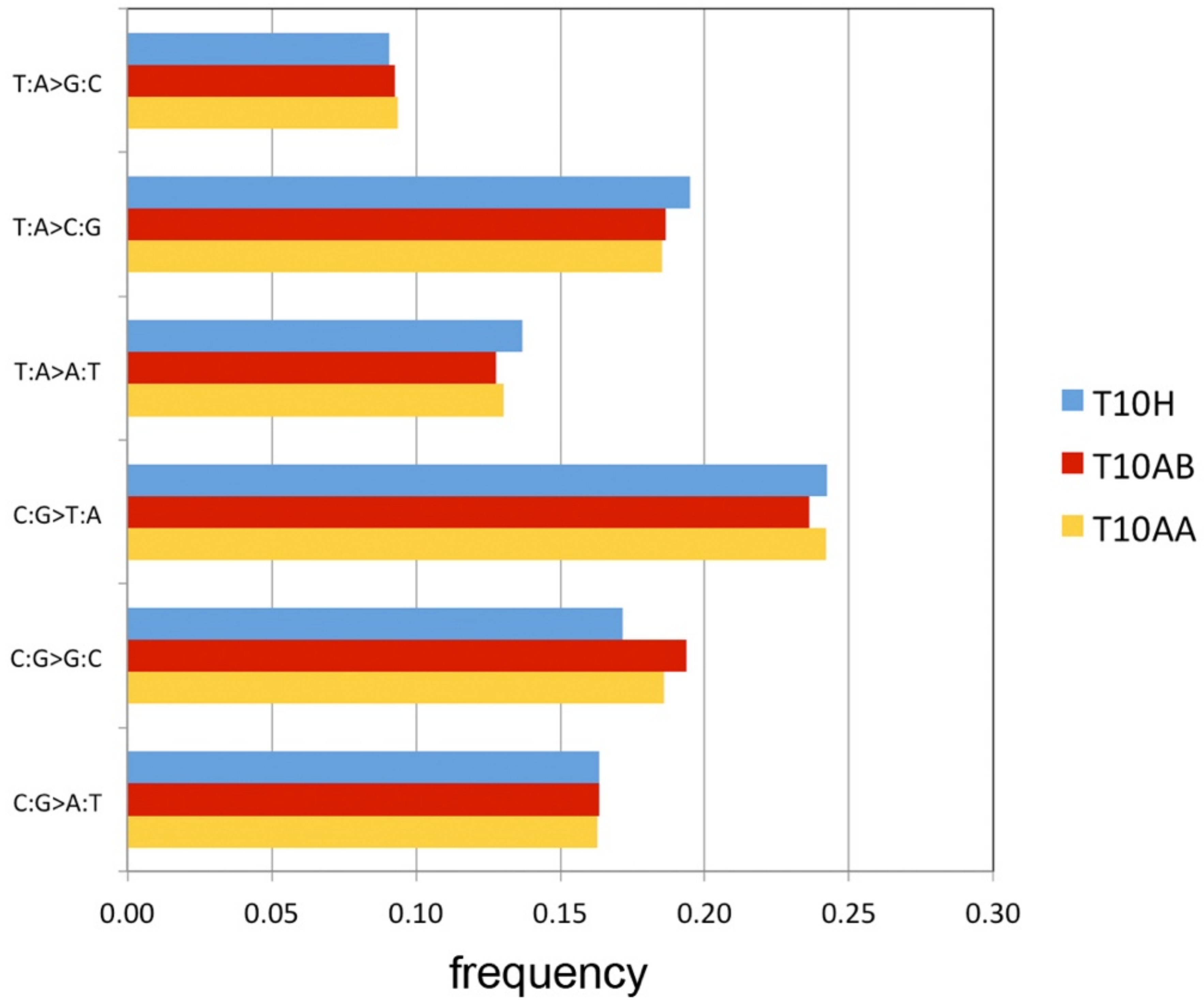

# Supplemental Figure 5

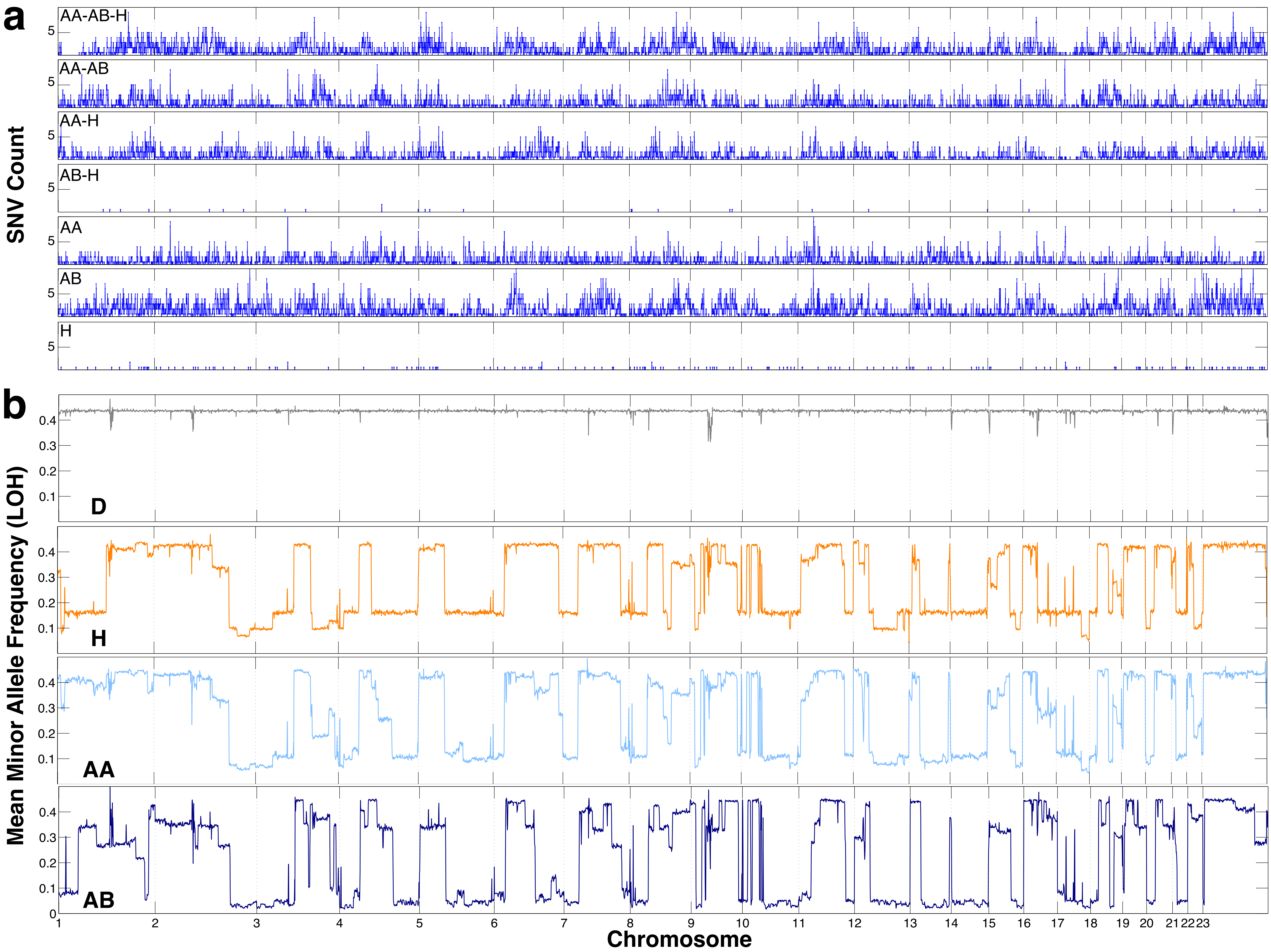

# Supplemental Figure 6

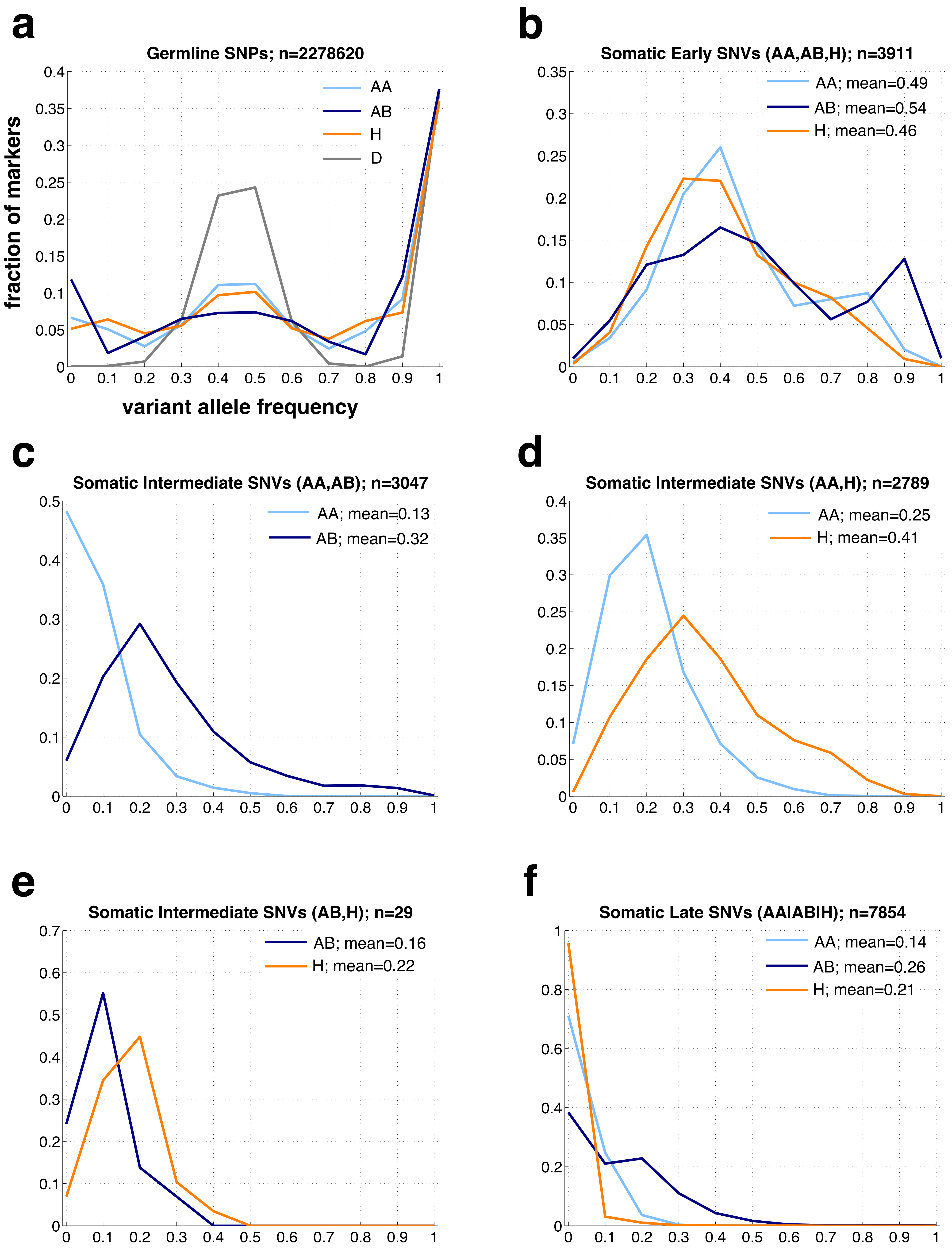

# Supplemental Figure 7

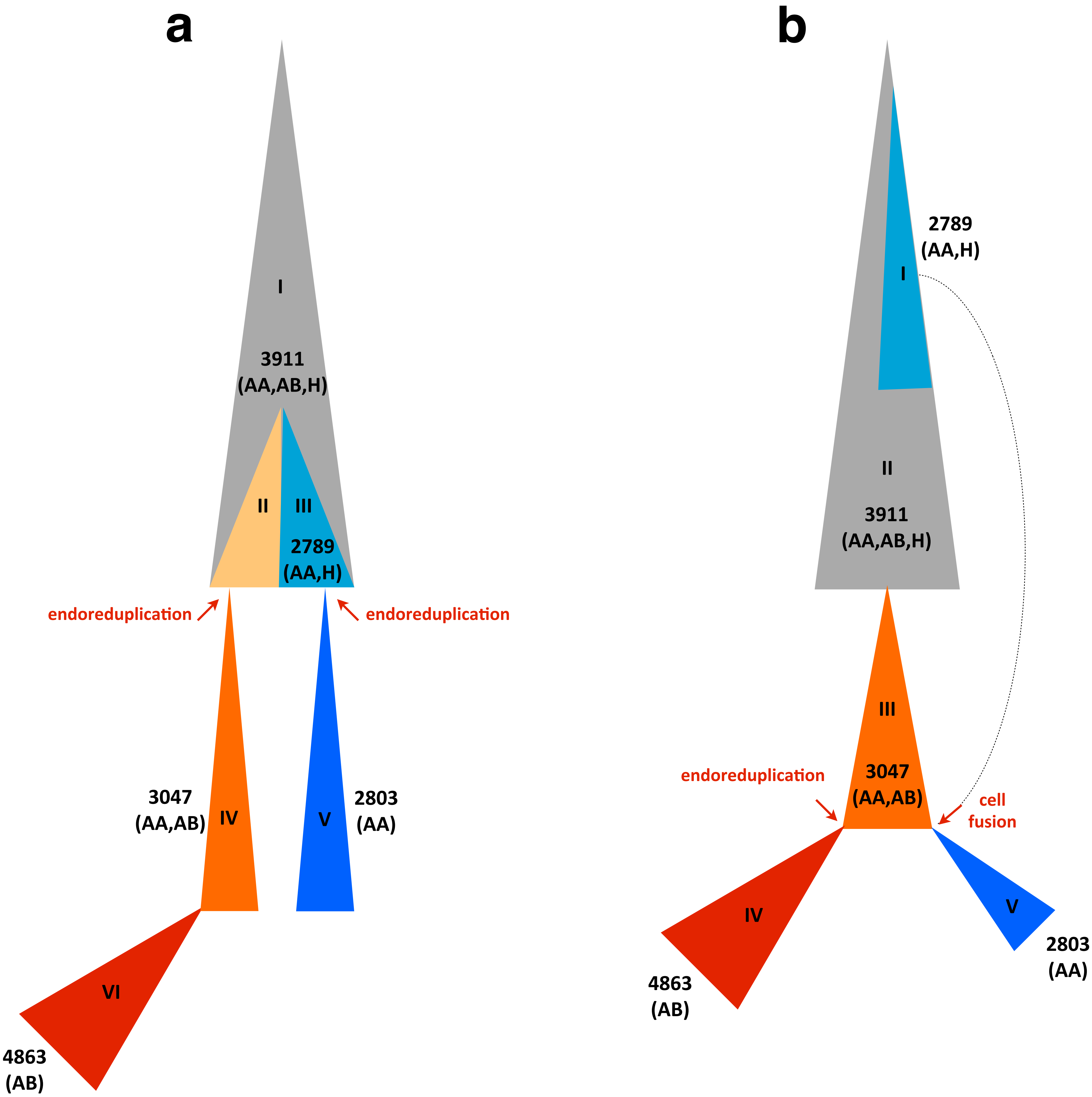

# Supplementary Figure 8

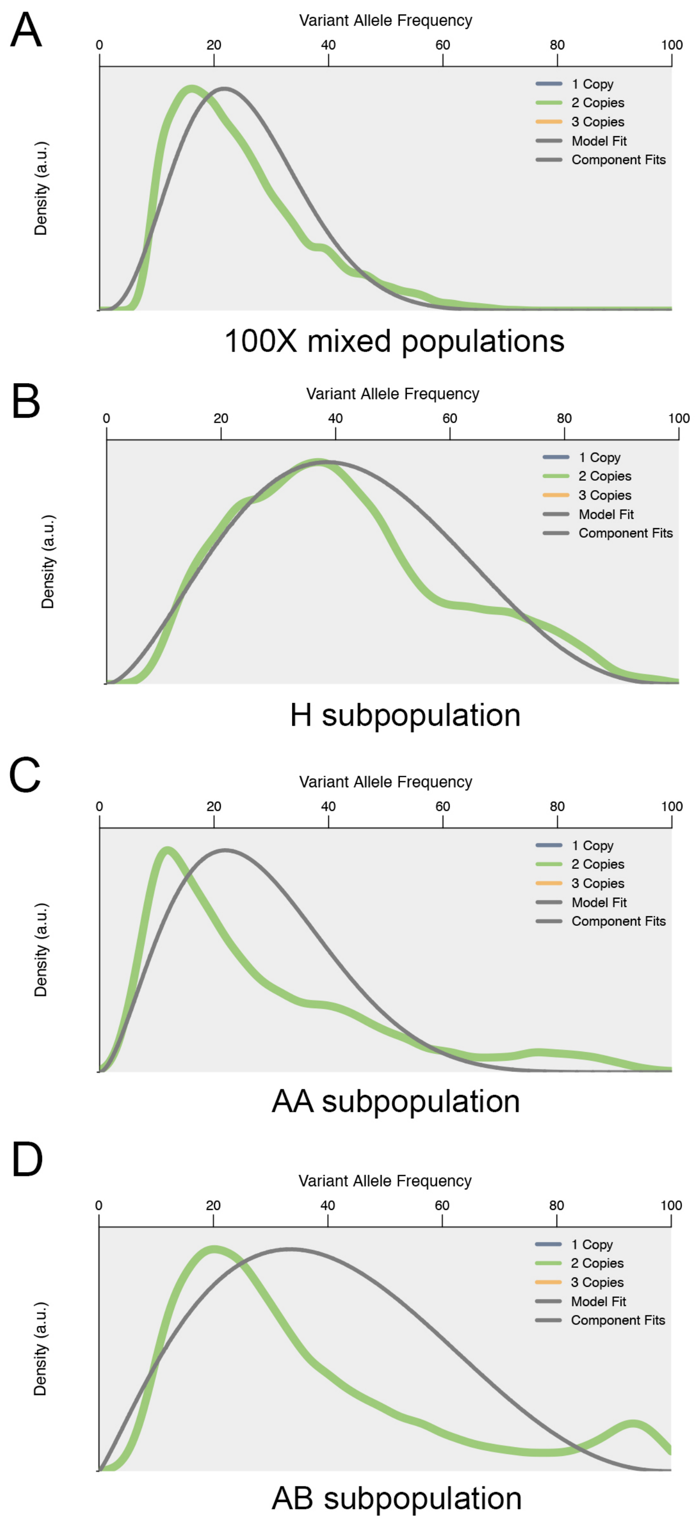

Supplementary Figure 9

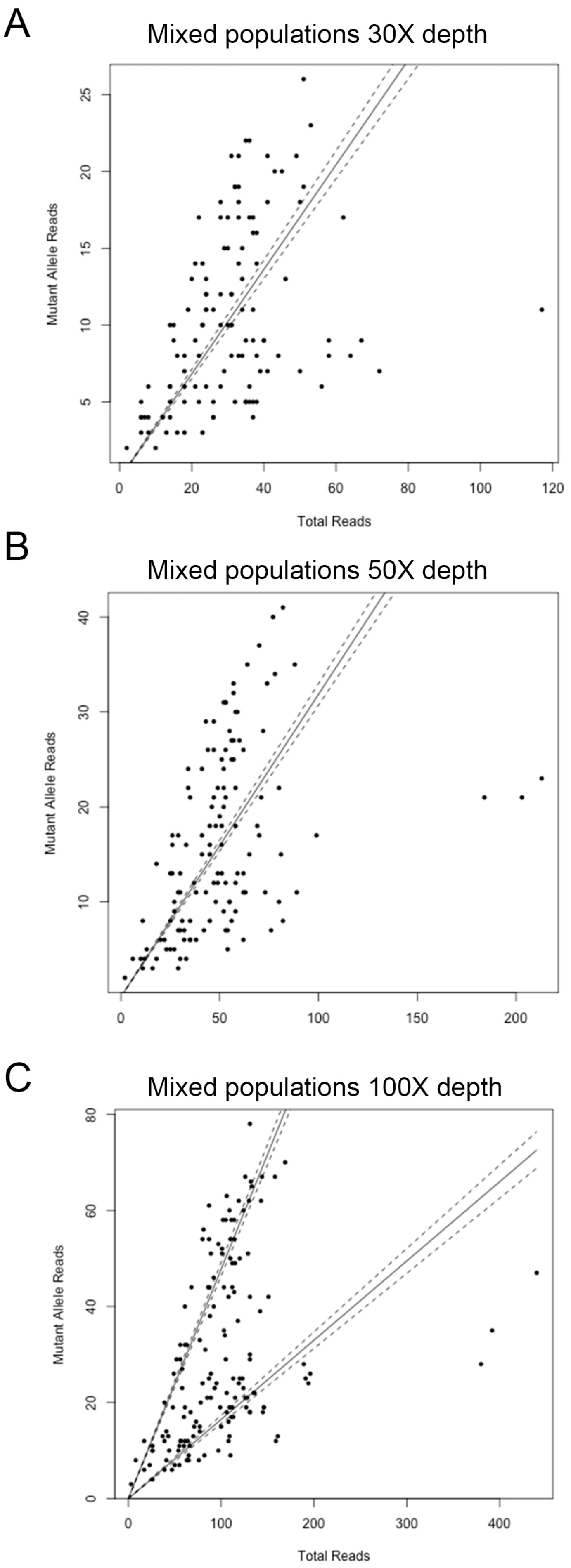

Supplement: Additional file 1: Figure S1. — Ploidy distributions of eight breast tumor samples. Figure S2. Data processing and variant detection pipeline. Figure S3. Structural variant detection and validation. Figure S4. Mutation spectrum frequencies in tumor subpopulations. Figure S5. Genome-wide LOH and SNV plots of tumor subpopulations. Figure S6. Tumor subpopulation allele frequency distributions. Figure S7. Evolutionary models of tumor progression. Figure S8. Inferring clonal substructure from mixed sequencing data and flow-sorted subpopulations using SciClone. Figure S9. Estimating tumor subpopulations from mixed sequencing data using PurBayes. [file 13073_2015_127_MOESM1_ESM.pdf]
