# Supplementary material for: Ploidy-Seq: inferring mutational chronology by sequencing polyploid tumor subpopulations
Source: Genome Med. 2015 Jan 28;7(1):6. doi: 10.1186/s13073-015-0127-5 (PMC4343275; doi:10.1186/s13073-015-0127-5)
Supplement: Additional file 2: Table S1. — Non-synonymous point mutations in tumor subpopulations. Table S2. Coding indels in tumor subpopulations. Table S3. Structural variants and copy number aberrations in genic regions. Table S4. LOH and genotype intersection table. [file 13073_2015_127_MOESM2_ESM.pdf]

SUPPLEMENTAL TABLE1 - NONSYNONYMOUS POINT MUTATIONS DETECTED IN TUMOR SUBPOPULATIONS

chr	start	stop	gene	REF	VAR	D	H	AA	AB	sub	CancerGe	Cosmic	POLYPHEN	SIFT	Nonsynonymous	GenBank	Exon	Nucleotide	AminoAcid
**EARLY SNVs**																			
1	184313967	184313968	HMCN1	G	C	GG	GC	GC	GC	H_AA_AB	NA	NA	0.991	0.06	nonsynonymous SNV	NM_031935	exon55	c.G8591C	p.R2864P
2	47891000	47891001	**FBXO11**	G	C	GG	GC	GC	GC	H_AA_AB	FBXO11	p.0?	0.995	0	nonsynonymous SNV	NM_001190274	exon19	c.C2297G	p.P766R
3	113368862	113368862	SLC9A10	G	T	GG	TG	TG	TG	H_AA_AB	NA	NA	0.949	0.03	nonsynonymous SNV	NM_183061	exon26	c.C3260A	p.T1087K
4	74529590	74529591	AFP	A	G	AA	AG	AG	AG	H_AA_AB	NA	NA	0.989	0	nonsynonymous SNV	NM_001134	exon7	c.A730G	p.S244G
6	37444241	37444242	RNF8	C	G	CC	CG	CG	CG	H_AA_AB	NA	NA	0.88	0	nonsynonymous SNV	NM_003958	exon3	c.C244G	p.L82V
7	98966928	98966929	ZKSCAN5	G	C	GG	CG	CG	CG	H_AA_AB	NA	NA	0.991	0.04	nonsynonymous SNV	NM_145102	exon7	c.G1640C	p.R547T
8	105430187	105430188	TM7SF4	T	A	TT	AT	AT	AT	H_AA_AB	NA	NA	0.984	0.06	nonsynonymous SNV	NM_030788	exon2	c.T231A	p.H77Q
8	146128603	146128603	ZNF16	C	G	CC	CG	CG	CG	H_AA_AB	NA	NA	0.932	0	nonsynonymous SNV	NM_006958	exon3	c.G374C	p.R125T
10	112339680	112339680	**SMC3**	G	C	GG	CG	CG	CG	H_AA_AB	SMC3	NA	0.187	0.16	nonsynonymous SNV	NM_005445	exon15	c.G1450C	p.A484P
11	61327496	61327496	FADS1	C	G	CC	GC	GC	GC	H_AA_AB	NA	NA	0.999	NA	nonsynonymous SNV	NM_013402	exon9	c.G1163C	p.S388T
11	64455499	64455499	**PPP2R5B**	G	A	GG	GA	GA	GA	H_AA_AB	PPP2R5B	NA	0.995	0	nonsynonymous SNV	NM_006244	exon9	c.G907A	p.V303M
11	88773158	88773159	**NOX4**	C	G	CC	CG	CG	CG	H_AA_AB	NOX4	na	0.999	0.03	nonsynonymous SNV	NM_001143837	exon13	c.G812C	p.C271S
12	21515202	21515202	RECQL	G	A	GG	GA	GA	GA	H_AA_AB	na	na	0.639856	na	nonsynonymous SNV	NM_032941	exon15	c.C1765T	p.Q589X
15	87823678	87823679	RHCG	A	C	AA	CA	CA	CA	H_AA_AB	NA	NA	0.826	0.01	nonsynonymous SNV	NM_016321	exon5	c.T716G	p.I239R
16	52061903	52061903	**RBL2**	C	G	CC	GC	GC	GC	H_AA_AB	RBL2	na	0.001	0.16	nonsynonymous SNV	NM_005611	exon16	c.C2353G	p.L785V
17	7518305	7518305	**TP53**	T	C	TT	TC	TC	TC	H_AA_AB	TP53	p.0?	1	0	nonsynonymous SNV	NM_001126114	exon7	c.A701G	p.Y234C
17	40284218	40284219	EFTUD2	T	C	TT	CT	CT	CT	H_AA_AB	NA	NA	0.819	0.08	nonsynonymous SNV	NM_004247	exon28	c.A2869G	p.M957V
19	56201777	56201777	KLK9	C	T	CC	CT	CT	CT	H_AA_AB	NA	NA	0.609	0.16	nonsynonymous SNV	NM_012315	exon3	c.G215A	p.R72H
19	59474623	59474623	LILRB2	G	T	GG	TG	TG	TG	H_AA_AB	NA	NA	0.609	0.17	nonsynonymous SNV	NM_001080978	exon6	c.C811A	p.Q271K
20	42180607	42180607	JPH2	T	A	TT	TA	TA	TA	H_AA_AB	NA	NA	0.815	0.01	nonsynonymous SNV	NM_020433	exon3	c.A1240T	p.I414F
20	45697833	45697834	**NCOA3**	G	T	GG	GT	GT	GT	H_AA_AB	NCOA3	NA	0.819	0.07	nonsynonymous SNV	NM_001174087	exon11	c.G1473T	p.K491N
20	58005192	58005193	**CDH26**	G	A	GG	GA	GA	GA	H_AA_AB	CDH26	NA	0.687	0.06	nonsynonymous SNV	NM_177980	exon13	c.G2000A	p.S667N
X	48256003	48256003	**PORCN**	A	G	AA	GA	GA	GA	H_AA_AB	PORCN	NA	0.997	0	nonsynonymous SNV	NM_203475	exon5	c.A638G	p.Y213C
**INTERMEDIATE H-AA**																			
2	45089300	45089300	SIX2	G	A	GG	GA	GA	GG	H_AA	NA	NA	0.999	0.01	nonsynonymous SNV	NM_016932	exon1	c.C454T	p.R152C
2	220065631	220065631	SPEG	G	A	GG	AA	GA	GG	H-AA	NA	NA	0.997	0.02	nonsynonymous SNV	NM_005876	exon41	c.G9683A	p.R3228H
2	153299787	153299787	ARL6IP6	C	G	CC	GC	GC	CC	H_AA	NA	NA	0.889	0	nonsynonymous SNV	NM_152522	exon3	c.C488G	p.A163G
2	165504215	165504215	SLC38A11	C	A	CC	AC	AC	CC	H_AA	NA	NA	0.983	0.01	nonsynonymous SNV	NM_173512	exon4	c.G294T	p.K98N
7	91956649	91956649	PEX1	C	T	CC	TC	TC	CC	H_AA	NA	NA	0.966	0	nonsynonymous SNV	NM_000466	exon23	c.G3661A	p.G1221R
7	99500036	99500036	ZSCAN21	A	G	AA	GA	GA	AA	H_AA	NA	NA	0.957	0.01	nonsynonymous SNV	NM_145914	exon4	c.A1282G	p.S428G
7	99634832	99634832	STAG3	C	A	CC	AC	AC	CC	H_AA	NA	NA	0.998	0	nonsynonymous SNV	NM_012447	exon15	c.C1479A	p.H493Q
8	107765681	107765681	OXR1	C	G	CC	GC	GC	CC	H_AA	NA	NA	0.985	0.07	nonsynonymous SNV	NM_018002	exon6	c.C443G	p.S148C
9	101030103	101030103	SEC61B	T	G	TT	GT	GT	TT	H_AA	NA	NA	0.954	0	nonsynonymous SNV	NM_006808	exon3	c.T203G	p.V68G
10	101649813	101649813	DNMBP	C	G	CC	GC	GC	CC	H_AA	NA	NA	0.997	0.04	nonsynonymous SNV	NM_015221	exon7	c.G2555C	p.G852A
11	13343659	13343659	ARNTL	C	G	CC	GC	GC	CC	H_AA	NA	NA	0.957	0	nonsynonymous SNV	NM_001030272	exon9	c.C496G	p.R166G
12	53050389	53050389	ZNF385A	G	C	GG	CC	CG	GG	H-AA	NA	NA	0.998	0.01	nonsynonymous SNV	NM_001130968	exon7	c.C836G	p.P279R
13	25865550	25865550	**CDK8**	A	C	AA	CA	CA	AA	H_AA	CDK8	NA	0.974	0.02	nonsynonymous SNV	NM_001260	exon7	c.A693C	p.E231D
19	18174416	18174416	**RAB3A**	G	T	GG	GT	GT	GG	H_AA	RAB3A	NA	0.795	0	nonsynonymous SNV	NM_002866	exon2	c.C135A	p.D45E
X	77044852	77044852	COX7B	T	A	TT	AT	AT	TT	H_AA	NA	NA	0.998	0.03	nonsynonymous SNV	NM_001866	exon2	c.T97A	p.F33I
**INTERMEDIATE AA-AB**																			
1	85346432	85346432	WDR63	G	T	GG	GG	GT	GT	AA_AB	NA	NA	0.996	0	nonsynonymous SNV	NM_145172	exon15	c.G1682T	p.W561L
2	55609611	55609611	CCDC104	C	T	CC	CC	CT	CT	AA_AB	NA	NA	0.723508	0.03	stopgain SNV	NM_080667	exon4	c.C376T	p.R126X
2	119632227	119632227	C1QL2	C	G	CC	CC	GC	GC	AA_AB	NA	NA	0.993	0.01	nonsynonymous SNV	NM_182528	exon1	c.G89C	p.R30P
2	218458742	218458742	TNS1	C	A	CC	CC	CA	CA	AA_AB	NA	NA	0.958	0.07	nonsynonymous SNV	NM_022648	exon13	c.G711T	p.E237D
4	79744486	79744486	ANXA3	G	C	GG	GG	GC	GC	AA_AB	NA	NA	1	0	nonsynonymous SNV	NM_005139	exon12	c.G821C	p.R274P
6	42017268	42017268	**CCND3**	C	G	CC	CC	GC	GC	AA_AB	CCND3	NA	0.155	0.07	nonsynonymous SNV	NM_001136125	exon1	c.G98C	p.R33P
9	138519119	138519119	**NOTCH1**	C	A	CC	CC	CA	CA	AA_AB	NOTCH1	p.A1611	0.98	0.08	nonsynonymous SNV	NM_017617	exon26	c.G4845T	p.M1615I
12	48029398	48029398	DNAJC22	C	T	CC	CC	TC	TT	AA_AB	NA	NA	0.881	0.02	nonsynonymous SNV	NM_024902	exon2	c.C476T	p.A159V
19	19488123	19488123	NDUFA13	C	T	CC	TT	CT	CC	AA_AB	NA	NA	0.533729	0.05	nonsynonymous SNV	NM_015965	exon1	c.C76T	p.P26S
X	39818786	39818786	**BCOR**	C	T	CC	CC	TC	TC	AA_AB	BCOR	NA	0.822	0	nonsynonymous SNV	NM_017745	exon4	c.G757A	p.V253I

X	49013974	49013974	PPP1R3F	C	G	CC	CC	GC	GC	AA_AB	NA	NA	0.997	0	nonsynonymous SNV	NM_033215	exon1	c.C698G	p.S233W
**LATE H**																			
10	73228920	73228920	CDH23	G	T	GG	TG	GG	GG	H	NA	NA	NA	0.02	nonsynonymous SNV	NM_001171933	exon4	c.G381T	p.W127C
14	74064993	74064993	LTBP2	G	C	GG	CG	GG	GG	H	NA	NA	0.992	0	nonsynonymous SNV	NM_000428	exon12	c.C2314G	p.P772A
22	36450653	36450654	**TRIOBP**	C	CC	TC	CC	CC		H	NA	NA	1	0.03	nonsynonymous SNV	NM_001039141		c.C3325A	p.V538G
**LATE AA**																			
3	135175310	135175310	SLCO2A1	A	T	AA	AA	TA	AA	AA	NA	NA	0.997	0	nonsynonymous SNV	NM_005630	exon3	c.T284A	p.V95E
4	151443356	151443356	LRBA	C	G	CC	CC	CG	CC	AA	NA	NA	0.98	0.01	nonsynonymous SNV	NM_001199282	exon53	c.G7888C	p.V2630L
16	29905282	29905282	TAOK2	C	T	CC	CC	CT	CC	AA	NA	NA	0.73533	0	stopgain SNV	NM_016151	exon16	c.C2188T	p.Q730X
16	69065993	69065993	FUK	C	T	CC	CC	CT	CC	AA	NA	NA	0.735252	0.04	stopgain SNV	NM_145059	exon17	c.C2050T	p.Q684X
21	29333268	29333268	USP16	G	A	GG	GG	GA	GG	AA	NA	NA	0.999	0.04	nonsynonymous SNV	NM_001032410	exon9	c.G783A	p.M261I
**LATE AB**																			
1	24013270	24013270	HMGCL	C	G	CC	CC	CC	GC	AB	NA	NA	1	0	nonsynonymous SNV	NM_000191	exon5	c.G494C	p.R165P
1	64911705	64911705	CACHD1	G	C	GG	GG	GG	CG	AB	NA	NA	0.882	0	nonsynonymous SNV	NM_020925	exon19	c.G2544C	p.Q848H
1	149206772	149206772	CERS2	G	C	GG	GG	GG	CG	AB	NA	NA	1	0	nonsynonymous SNV	NM_022075	exon6	c.C511G	p.P171A
1	158423091	158423091	ATP1A4	T	G	TT	TT	TT	TG	AB	NA	NA	0.001	0	nonsynonymous SNV	NM_001001734	exon5	c.T476G	p.V159G
1	202679223	202679223	**PIK3C2B**	T	C	TT	TT	TT	CT	AB	PIK3C2B	NA	0.002	0.36	nonsynonymous SNV	NM_002646	exon20	c.A2993G	p.N998S
1	203540220	203540220	NUAK2	G	C	GG	GG	GG	CG	AB	NA	NA	1	0	nonsynonymous SNV	NM_030952	exon7	c.C868G	p.R290G
2	8837329	8837329	KIDINS220	G	T	GG	GG	GG	TG	AB	NA	NA	0.999	0.03	nonsynonymous SNV	NM_020738	exon18	c.C2296A	p.Q766K
2	27163654	27163654	KHK	G	C	GG	GG	GG	GC	AB	NA	NA	0.996	0.03	nonsynonymous SNV	NM_000221	exon1	c.G23C	p.C8S
2	31423213	31423213	XDH	C	A	CC	CC	CC	AC	AB	NA	NA	0	0.04	nonsynonymous SNV	NM_000379	exon30	c.G3277T	p.A1093S
2	43955913	43955913	ABCG8	T	G	TT	TT	TT	TG	AB	NA	NA	0.722	0.08	nonsynonymous SNV	NM_022437	exon11	c.T1613G	p.V538G
2	196367387	196367387	DNAH7	C	A	CC	CC	CC	AC	AB	NA	NA	0.999	NA	nonsynonymous SNV	NM_018897	exon57	c.G10636T	p.G3546C
2	241914187	241914187	SEPT2	C	A	CC	CC	CC	AC	AB	NA	NA	1	0	nonsynonymous SNV	NM_006155	exon3	c.C116A	p.T39K
6	158436929	158436929	SYNJ2	C	A	CC	CC	CC	CA	AB	NA	NA	0.567	0	nonsynonymous SNV	NM_001178088	exon26	c.C3325A	p.L1109M
7	116320322	116320322	**CAPZA2**	G	T	GG	GG	GG	TG	AB	CAPZA2	NA	0.735424	0.04	stopgain SNV	NM_006136	exon3	c.G142T	p.E48X
8	12627648	12627648	**LONRF1**	G	A	GG	GG	GG	GA	AB	LONRF1	NA	0.735526	0.03	stopgain SNV	NM_152271	exon11	c.C2122T	p.Q708X
8	124273249	124273249	FAM83A	T	C	TT	TT	TT	CT	AB	NA	NA	1	0	nonsynonymous SNV	NM_032899	exon2	c.T506C	p.F169S
9	139057259	139057259	NPDC1	A	C	AA	AA	AA	CA	AB	NA	NA	0.981	0	nonsynonymous SNV	NM_015392	exon2	c.T200G	p.L67R
10	126681614	126681614	CTBP2	G	A	GG	GG	GG	AG	AB	NA	NA	0.982	0	nonsynonymous SNV	NM_001329	exon5	c.C263T	p.T88I
11	36475355	36475355	TRAF6	C	T	CC	CC	CC	TC	AB	NA	NA	1	0	nonsynonymous SNV	NM_145803	exon5	c.G485A	p.C162Y
12	6897561	6897561	ENO2	C	T	CC	CC	CC	TC	AB	NA	NA	0.997	0	nonsynonymous SNV	NM_001975	exon7	c.C641T	p.A214V
16	28748729	28748729	ATXN2L	C	T	CC	CC	CC	CT	AB	NA	NA	0.996	0	nonsynonymous SNV	NM_148416	exon8	c.C883T	p.R295C
17	71582716	71582716	GALR2	G	T	GG	GG	GG	TG	AB	NA	NA	0.721	0.06	nonsynonymous SNV	NM_003857	exon1	c.G157T	p.G53C
17	76537879	76537879	**RPTOR**	G	T	GG	GG	GG	TG	AB	RPTOR	NA	0.735446	0.09	stopgain SNV	NM_020761	exon28	c.G3307T	p.E1103X
18	20310787	20310787	HRH4	G	A	GG	GG	GG	GA	AB	NA	NA	0.883	0.04	nonsynonymous SNV	NM_021624	exon3	c.G436A	p.V146M
19	10999535	10999535	**SMARCA4**	C	G	CC	CC	CC	GC	AB	NA	p.?	0.999	0	nonsynonymous SNV	NM_001128845	exon23	c.C3291G	p.H1097Q
19	50546058	50546058	KLC3	G	C	GG	GG	GG	CG	AB	NA	NA	0.862	0	nonsynonymous SNV	NM_177417	exon12	c.G1386C	p.K462N
22	31267569	31267569	SYN3	T	G	TT	TT	TT	GT	AB	NA	NA	0.788891	0	nonsynonymous SNV	NM_001135774	exon8	c.A902C	p.Y301S

SUPPLEMENTAL TABLE 2 - INDELS DETECTED IN THE CODING REGIONS OF TUMOR SUBPOPULATIONS

chrom	position	gene	REF	VAR	Sub	Cancer	mut_class	genbank	exon	nucleotide	amino_acid
chr22	36813121	BAIAP2L2	A	insCATGGAGGT	H_AA_AB	NA	nonframeshift insertion	NM_025045	exon11	c.1215_1216ins	p.S406delins
chr11	76573419	MYO7A	G	del22bp	H_AA_AB	NA	frameshift deletion	NM_001127179	exon27	c.3514_3535del	p.1172_1179del
chr1	1.2E+08	ADAM30	C	del_C	AA_AB	NA	frameshift deletion	NM_021794	exon1	c.2290delG	p.A764fs
chr19	12997227	NFIX	C	del_C	AA_AB	NA	frameshift deletion	NM_001271043	exon2	c.444delC	p.I148fs
chr11	65062348	SCYL1	G	del_GGGA	AA_AB	NA	frameshift deletion	NM_001048218	exon17	c.2228_2231del	p.743_744del

Supplemental Table 3 - Structural Variants and Copy Number Abberations in Tumor Subpopulations

Type	chrA	chrA_start	chrB	chrB_start	Gene Name(s)	Sub	size (bp)	region	Method
EARLY SVs									
INS	chr11	100480573	chr11	100472903	PGR	AA,AB,H	7670	intronic	CREST
INS	chr17	56362930	chr17	56350448	BCAS3	AA,AB,H	12482	intronic	CREST
DEL	chr13	38984211	chr13	38984285	LHFP	AA,AB,H	74	intronic	SRM
DEL	chr9	20353001	chr9	20354059	MLLT3	AA,AB,H	1058	exon5	SRM
INTERMEDIATE SVs									
DEL	chr20	39145037	chr20	39145097	TOP1	AA,AB	60	intron	SRM
DEL	chr21	35085983	chr21	35131642	RUNX1	AA,AB	45659	3'UTR	SRM
DEL	chr3	157111517	chr3	157112834	GMPS	AA,AB	1317	intron	SRM
DEL	chr7	92172871	chr7	92173002	CDK6	AA,AB	131	intron	SRM
DEL	chr14	55202458	chr14	55202509	KTN1	AA,AB	51	intron	SRM,CREST
DUP	chr7	137885296	chr7	137908932	TRIM24	AA,AB	23636	intron	SRM
INTRA	chr14	91560812	chr14	99049952	TRIP11:C14orf65	AA,AB	na	exon	SRM,CREST
INS	chr9	92605259	chr9	85556060	SYK	AB,H	7049199	intron	CREST
INTER	chr10	123338198	chr11	124189007	FGFR2:BC036195	AB,H	na	intron	SRM
LATE SVs									
DEL	chr1	143649461	chr1	143649664	PDE4DIP	AA	203	intron	CREST
DEL	chr8	128818158	chr8	128819005	MYC	AB	847	intron	CREST
INS	chr10	22068850	chr10	21214251	MLLT10	AB	854599	intron	CREST
DEL	chr22	39230128	chr22	39230185	MKL1	AB	57	intron	SRM
AMP	chr12	22072826	chr12	26085603	KRAS ++	AB	4012777	genes	VARBIN
DEL	chr5	101715241	chr5	109773692	EFNA5 ++	AB	8058451	genes	VARBIN
DEL	chr19	1279911	chr19	4634233	COL5A ++	AB	3354322	genes	VARBIN

Supplemental Table 4 - Overlap between SNVs and LOH blocks

		LOH Genotype							
SNV Genotype		**AA, AB, H**	**AA, AB**	**AA, H**	**AB, H**	**AA**	**AB**	**H**	**None**
	**AA, AB, H (n=3911)**	22.0%	0.0%	2.6%	0.2%	0.0%	4.7%	3.9%	66.6%
	**AA, AB (n=3047)**	23.2%	0.0%	6.3%	1.8%	0.0%	4.9%	10.1%	53.8%
	**AA, H (n=2789)**	23.7%	0.1%	3.0%	2.2%	0.0%	9.2%	3.1%	58.7%
	**AB,H (n=29)**	24.1%	0.0%	0.0%	0.0%	0.0%	0.0%	10.3%	65.5%
	**AA (n=2803)**	37.4%	0.0%	2.9%	1.5%	0.0%	5.5%	7.3%	45.4%
	**AB (n=4863)**	24.4%	0.0%	3.8%	0.9%	0.0%	3.8%	5.9%	61.2%
	**H (n=188)**	33.0%	0.0%	1.1%	0.5%	0.0%	8.0%	2.7%	54.8%
	**Expected (n=17630)**	38.1%	0.1%	2.8%	1.9%	0.0%	4.5%	6.5%	46.1%
